# Salivary microbiome and periodontopathogen/denitrifying bacteria associated with gingivitis and periodontitis in people with type 2-diabetes

**DOI:** 10.12688/f1000research.161731.1

**Published:** 2025-03-14

**Authors:** Endang Bachtiar, Boy M. Bachtiar, Dicky L Tahapary, Turmidzi Fath, Citra Fragrantia Theodora, Natalina Haerani, Selvi Nafisa Shahab, Yuniarti Soeroso, Ardy Wildan, Fergie Marie Joe Grizella Runtu, Fatimah Maria Tadjoedin, Dewi Ayuningtyas

**Affiliations:** 1 Oral Biology Fac. of Dentistry, University of Indonesia, Depok, West Java, 16424, Indonesia; 2 Clinical Research Unit RSCM, . Metabolic-Endocrine-Diabetes Division, Dept. Internal Medicine, Universitas Indonesia, Depok, West Java, Indonesia; 3 Faculty of Dentistry, Department of Oral Biology and Oral Science Research Center, Universitas Indonesia, Depok, West Java, 10430, Indonesia; 4 Faculty of Dentistry,Department of Periodontology, Universitas Indonesia, Jakarta, Jakarta, 10430, Indonesia; 5 Faculty of Medicine, Department Microbiology, Clinical Research Unit RSCM, Universitas Indonesia, Ciptomangunkusumo Hospital., West Java, Indonesia; 6 Metabolic-Endocrine-Diabetes Division, Dept. Internal Medicine, Universitas Indonesia, Depok, West Java, Indonesia

**Keywords:** Diabetes; gingivitis; periodontitis; salivary microbiome; nanopore sequencing

## Abstract

**Background:**

Despite diabetes mellitus and periodontal diseases are mutually exclusive, little is known about particular types of bacteria that may have exacerbated the development of diabetics’ periodontal inflammation. This study’s aim was to compare the salivary microbiomes of individuals with type 2 diabetes (20–40 years old) who had gingivitis or periodontitis to those who did not. Additionally, we evaluated the relationship between the number of periodontopathogens and the amount of nitrate-reducing bacteria in their salivary microbiome.

**Methods:**

Saliva was collected, DNA was isolated, the entire 16S ribosomal RNA gene was amplified, and sample libraries were prepared in accordance to the Oxford Nanopore MinION Technology procedure. The relative abundance and bacterial diversity in saliva samples that were pooled according to three groups; T2DM patients without periodontal disease (G1), T2DM patients with gingivitis (G2), and T2DM patients with periodontitis (G3), was measured using bioinformatic methods. Additionally, the relationships between the periodontopathic bacteria ( *Porphyromonas gingivalis*, *Treponema denticola*, *Tannerella forsythia*, and *Fusobacterium* spp.) and denitrifying community ( *Haemophilus*, *Neisseria*, *Rothia*, and *Veillonella*) were assessed.

**Results:**

Alpha-diversity analysis revealed, the G1 group had significantly lower bacterial diversity and abundance than groups G2 and G3 (p< 0.0001). However, the microbiota profiles of diabetic patient groups with periodontitis and gingivitis were comparable. Using receiver operating characteristic (ROC) analysis, potential biomarkers for differentiating between gingivitis and periodontitis were discovered. Areas under the curve (AUC) between *Fusobacterium* spp. and *Neisseria* were found to be 0.94 (p = 0.43), while the AUC between *P. gingivalis* and *Rothia* was not significant (0.84, p = 0.08).

**Conclusion:**

People with type 2 diabetes mellitus who also have gingivitis or periodontitis exhibit different relationships between periodontopathic and denitrifying bacteria in their salivary microbiome. These features might be essential indicators for early identification and treatment of gingivitis in order to prevent periodontitis.

## Introduction

The development of periodontal disease is known to be significantly influenced by the oral microbiota, which may also be an important variable in systemic disorders including diabetes and heart disease. ^1^ Additionally, recent studies indicate that those with type 2 diabetes (T2DM) are more likely to develop periodontitis and to have more severe forms of disease. ^2– 4^ Even though numerous studies have looked at the bidirectional relationship between diabetes mellitus and periodontal disorders, there are still gaps in the quantity and quality of study on the subject. ^5, 6^

The most common types of periodontal disease are gingivitis and periodontitis. In many respects, gingivitis is a state of stable inflammation that represents equilibrium (homeostasis). Hence, gingivitis is mostly reversible. ^7^ However, the prolonged or recurrent occurrence of the condition, particularly in predisposed individuals, may lead to irreversible destruction of hard and soft tissues (periodontitis). Both gingivitis and periodontitis have been recognized as polymicrobial, biofilm-based inflammatory diseases ^8^ in which a disruption of the ecological balance between the biofilm and the periodontal tissue homeostasis plays a more important role than specific infections, ^9– 11^ alongside other environmental, lifestyle and genetic risk factors.

In patients with periodontitis, the number of periodontopathogens ( *Porphyromonas gingivalis*, *Treponema denticola*, and *Tannerella forsythia*) and *Fusobacterium* spp. is increasing, while the number of normal flora bacteria is decreasing. ^12– 14^ Healthy microbial populations include all genera; *Rothia, Neisseria, Actinomyces, Veillonella, Kingella*, *Propionibacterium*, *Prevotella, Granulicatella,* and *Haemophilus.* They are all recognized to be nitrate-reducing bacteria (NRB), ^15, 16^ and have antimetabolic disease activities. ^17^ These NRB have attracted much attention currently given their crucial function in the human nitrogen cycle’s nitrate (NO3-) - nitrite (NO2-) - nitric oxide (NO) pathway. ^18, 19^ Additionally, blood pressure regulation and insulin resistance are positively impacted by oral nitrate-reducing bacteria. ^20^ Therefore, the purpose of this study was to evaluate both the composition of the oral microbiota in diabetic individuals with and without periodontal disease, along with the relationship between the periodontopathic and the NRB, with a particular emphasis on *Neisseria*, *Veillonella*, *Haemophilus*, and *Rothia.* Saliva was chosen as the sample type to accurately capture the oral microbiome’s composition, since it is commonly used to explain the microbial shifts that occur in periodontal sites as they progress from health to disease. ^21– 24^ Furthermore, the Oxford Nanopore Technology (ONT) long-read sequencing approach was performed to identify taxa based on whole 16S rRNA sequences, which allows the species-level identification of the representative microbes, including periodontopathogen ^25^ and oral bacteria associated with nitrate-reducing. ^26^

## Methods

### Participants and patient characteristic

Participants in this cross-sectional study were Indonesian adult patients recruited from the Dr. Cipto Mangunkusumo Hospital in Jakarta between November 2023 and January 2024. Saliva was collected from 171 participants who met the inclusion and exclusion criteria. The first criteria was 20-40 years old with type 2 diabetes (T2DM, non-insulin-dependent diabetes) diagnosed by the internist of the Division of Endocrinology, based on the conditions of blood glucose level 2 hours after oral glucose load **≥** 200 mg/dL, HbA1c ≥ 6.5%, or plasma glucose was ≥ 200 mg/dl with classic hyperglycemic crisis. In addition, all participants fulfilled the following conditions: 1) did not use antibiotics or nonsteroidal anti-inflammatory drugs and did not smoke within three months prior to saliva collection; 2) had at least 20 teeth at the time of sample collection; 3) had no overt symptoms of oral mucosal or root caries; 4) had not undergone periodontal surgery or therapy in the previous six months; 5) did not consume any food for at least one hour prior to sample collection. Patients with following conditions were excluded from the study: 1) diagnosed with a metabolic disorder other than diabetes such as hyperthyroidism or cancer that could have influenced the development of the periodontitis; 2) used certain medication such as hormones within six months prior to the sample collection; 3) were pregnant or breastfeeding.

Participants were further divided into three groups: diabetic patients without evidence of periodontal diseases (Group 1, n = 28); diabetic patients with gingivitis (Group 2, n = 54); diabetic patients with periodontitis (Group 3, n = 89). Age (20-40 years old) and sex (35–50% male) were balanced across the three groups. The periodontal evaluations were carried out by competent periodontists. The diagnosis of gingivitis was made by bleeding on probing (BOP) score, ^27^ while chronic periodontitis was diagnosed based on the standard classification of the American academic of periodontology. ^28^ Participants diagnosed with chronic periodontitis were identified as having at least 30% of sites with alveolar bone resorption, as well as more than 4 sites with probing depth (PD) ≥ 4 mm and clinical attachment loss (CAL) ≥ 2 mm.

In accordance with the criteria of the institutional ethics committee, All participants provided written informed consent before they participated part in the study, and the Dr. Cipto Mangunkusumo Hospital’s Ethics Committee approved the study’s protocols (Ethics Reference Number: KET-1203/UN2.F1/ETIK/PPM.00.02/2023).

### Saliva sample collection, DNA extraction, and sequencing

Participants’ saliva samples (3-5 mL) were collected according to procedure reported elsewhere ^29^ one hour after they refrained from eating, drinking, or brushing their teeth. After rinsing their mouths, the participants immediately spit 3–5 mL of unstimulated whole saliva into a 25 mL conical tube. Samples were kept at -80°C until DNA extraction. DNA was extracted using the Monarch ^®^^,^^™^ Genomic DNA purification kit, NEB #T3010S/L (New England Biolabs, Bruningstrasse Frankfurt am Main, Germany) and quantified using a Qubit 2.0 Fluorometer (Invitrogen, Carlsbag, CA, USA). Following PCR amplification, PCR products from each sample were purified and quantified using the Qubit. ^30^ The genomic DNA of each study group’s samples was ligated in equimolar quantities to create a pool of three group libraries for nanopore sequencing. Each barcoding kit contained 50 ng of starting DNA. Nanopore amplicon library was prepared using the 16S Barcoding Kit 24 V14 (SQK-RAB204, Oxford Nanopore Technologies, UK) following the manufacturer’s instruction. The kit contains primer 27F and 1492R for amplification of the full-length 16S rRNA gene. Each barcoding kit contained 50 ng of starting DNA.

Sequencing was conducted using the MinION (Oxford Nanopore Technologies, UK) with a MinION flow cell (R10.4.1) for 8 hours. ^25^ Subsequently, the basecalling were generated using MinKNOW (Oxford Nanopore Technologies, UK). For microbiota profiling analysis, we followed the EPI2ME for wf-metagenomic workflow for real time analysis. The analysis results were further generated in the form of a report in the EPI2ME for wf-metagenomic. ^25^

### Measuring the nitrite and nitrate content in saliva

Nitrate and nitrite levels in unstimulated saliva samples were quantified using the Griess Reagent System, Promega #TB229, Madison, WI 53711-5399 USA). ^20^ The procedure involved mixing 50 μL of the supernatant with 50 μL of Griess reagents, allowing the mixture remain at room temperature for 10 minutes in the dark, and then using a spectrophotometer (AccuReader. M965/M965+, Nangang, Taipei, Taiwan), to quantify the mixture at 450 nm.

### Bioinformatic and statistical analysis

For microbiota profiling analysis (OTU, alpha diversity, and rarefaction curve), the EPI2ME for wf-metagenomic was used. To endure data quality, only high-quality reads (“pass”, >5 cumulative reads) were included in the analysis. ^31^ The operational taxonomic units (OTUs) in each group and the rarefaction curve were developed using EPI2ME, and analysed using RStudio 4.3.3. The software was also used to assess alpha diversity (representing the taxonomic abundance profiles) between groups, which was examined using the Simpson and Shannon indices (representing species evenness and richness). We used the Kruskal-Wallis test to evaluate the groups’ differences in alpha diversity, while statistical difference of the comparing between two groups (G1/G2 and G3) was determine by Student’s t-tests and that among all groups (G2, G3, and G3) by one-way ANOVA test followed by Barlet’s test. The interaction between periodontopathic bacteria and NRB as a predictor of the development of periodontal damage in diabetic subjects was also evaluated for sensitivity and specificity using the receiver operating characteristic (ROC) approach. All statistical analyses were conducted using GraphPad Prism software version 10 for Mac, which is licensed and requires an annual subscription (GraphPad Software, San Diego, CA, USA). A significance level of p< 0.05 was employed. R Studio is an alternative to GraphPad for statistical analysis.

## Results

### Read analysis, the rarefaction curve and alpha diversity of each group’s microbiota

**Figure 1.**
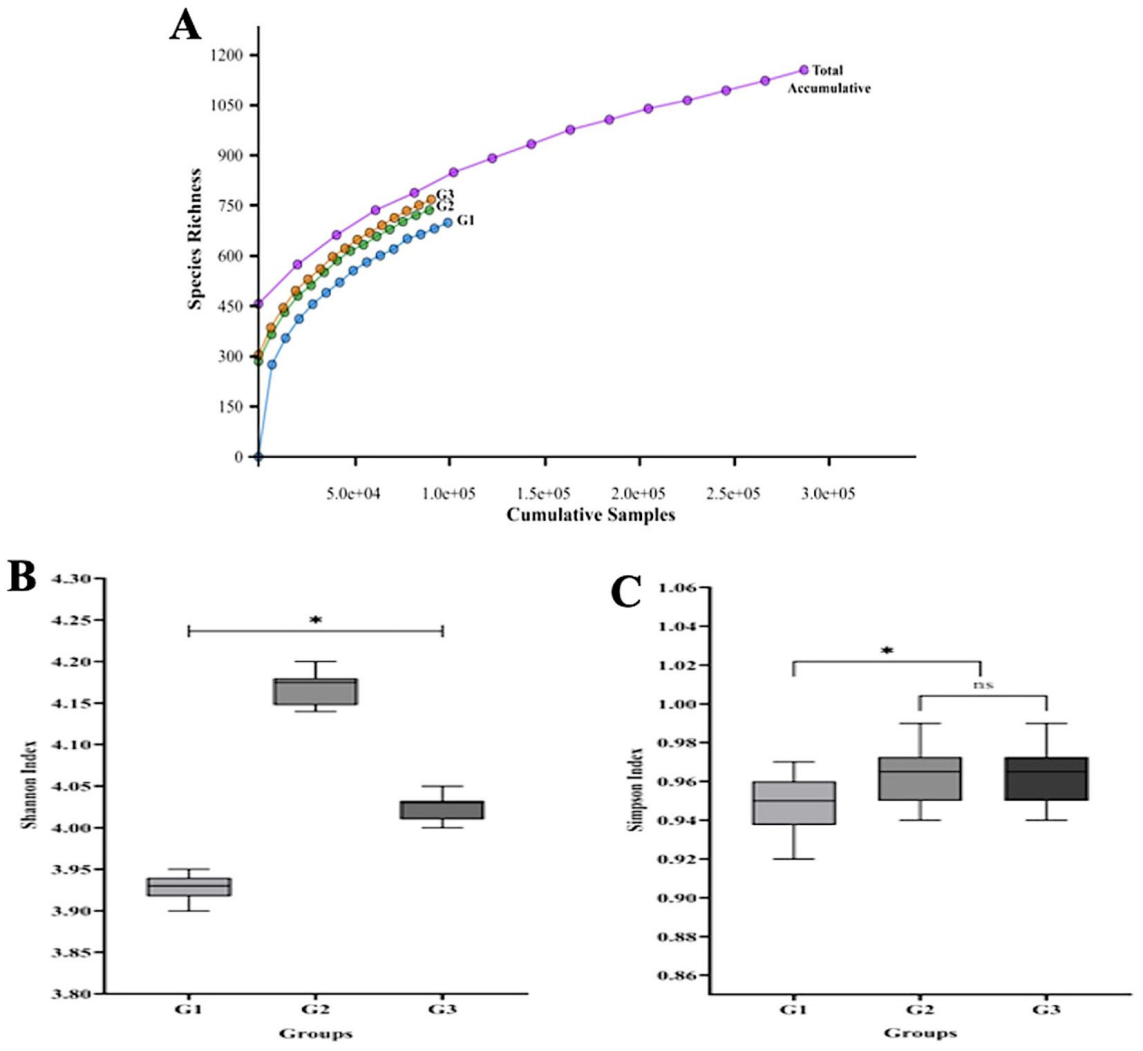
The salivary microbiome's rarefaction curve and alpha diversity among different T2DM patient groups. The total number of sequences is displayed on the horizontal axis, while the number of operational taxonomic units (OTUs) at a 97% intersequence similarity level is shown on the vertical axis of the rarefaction curve (A). The diversity indices of Shannon (B) and Simpson (C) show how alpha diversity varies among the three groups in terms of evenness and richness. Species diversity and evenness seem to be larger in gingivitis group (G2) and periodontitis group (G3) compared to oral health group (G1). Asterisk (*) indicates a statistically significant difference (p-value < 0.05).

As shown in Figures 1A,B,C, the majority of the 16S rRNA gene’s V1-V9 hypervariable regions were covered by the 287,215 sequence reads that were produced. Each read in this study was a unidirectional base calling sequence (i.e. it was either forward or backward). A shift in the microbial composition between the groups was shown by rarefaction curves and alpha diversity indices (Shannon and Simpson). There are significant variations in the overall bacterial diversity between G1, G2, and G3. According to the Shannon index, the gingivitis (G2 group) showed a higher relative abundance of bacteria (p< 0.05) than either the periodontitis (G3 group) or oral health (G1 group). However, the Simpson index revealed that the species richness of groups G2 and G3 was comparable.

### Bacterial variability comparison between T2DM patient groups

**Figure 2.**
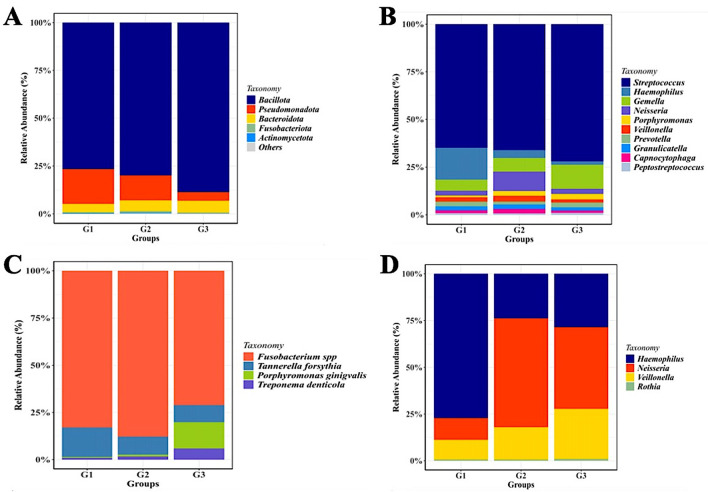
Saliva microbiota taxonomic composition in T2DM patients with and without periodontal diseases. Relative abundance of mayor of salivary bacterial taxa, which relative abundance >5%, are presented at the phylum (A) and genus (B) level. “Others” refers to the remaining phyla. A comparison of the relative abundance of periodontopathic bacterial species (C) and genus of NO _3_^-^-reducing bacteria (D) between three groups of patient with T2DM. G1 through G3 represent the participant group.

Operational taxonomic unit (OTU) clustering was performed and a total of 1123 OTUs were obtained with 97% identity of coverage for each group. As shown by the histogram in Figures 2A, the phylum-level compositions of the G1 and G2 groups are comparable. However, *Bacillota* ( *Firmicutes*) consistently represent the dominant phyla in all groups, although their relative abundance in the G2 and G3 groups appears to be slightly lower than in the G1 group. Next the most prevalent phyla were *Pseudomonadota* ( *Proteobacteria*), although their numbers were lower in the G3 group than in the G1 and G2 groups. *Bacteroidota* ( *Bacteroides*) came next, with almost equal numbers in each group, and the prevalence other phyla was less than 5%.

Although the phylum-level microbiome is dominated by *Firmicutes*, the relative abundance of certain genera varies significantly amongst the groups. *Streptococcus* was the most prevalent taxa in each group. *Haemophilus* was most prevalent in group G1 (14%), followed by groups G2 and G3 (3% and 1.5%, respectively). *Neisseria* (22%) and *Gemella* (24%) were two other prominent genera that were shown to be abundant in the G2 and G3 groups, respectively. Although the three groups’ relative abundances of *Porphyromonas* seem to vary, group G3 still has higher numbers of these genera than the other two groups ( Figure 2B).

### Comparative abundance of salivary periodontopathogens and nitrate-reducing bacteria based on the pooled samples’ 16S rRNA gene sequencing

Periodontopathic and nitrate-reducing bacteria in each of the patient groups were the focus of an expanded evaluation of changes in the salivary microbiome. Figure 2C shows that *T. forsythia* was more prevalent in the saliva of diabetic individuals without periodontal diseases (G1), whereas *Fusobacterium* spp. were the bacteria that were found in higher proportion in the saliva of all groups under study (G1, G2, and G3) (p<0.005). Although *P. gingivalis* was present in all groups, the G3 group exhibited a greater amount of this species than the other groups. In terms of the nitrate-reducing bacteria ( *Haemophilus*, *Rothia*, *Veillonella*, and *Neisseria*), the G1 group had a considerably higher level of *Haemophilus* than the G2/G3 group (p< 0.05). *Rothia* proportions were consistently lower in all groups than *Veillonella*, whose numbers increased gradually from the G1-G2 and G3 groups. The proportion of *Neisseria* was significantly lower in the G1 group than in the G2 and G3 groups, where the bacterial counts were comparable ( Figure 2D).

**Figure 3.**
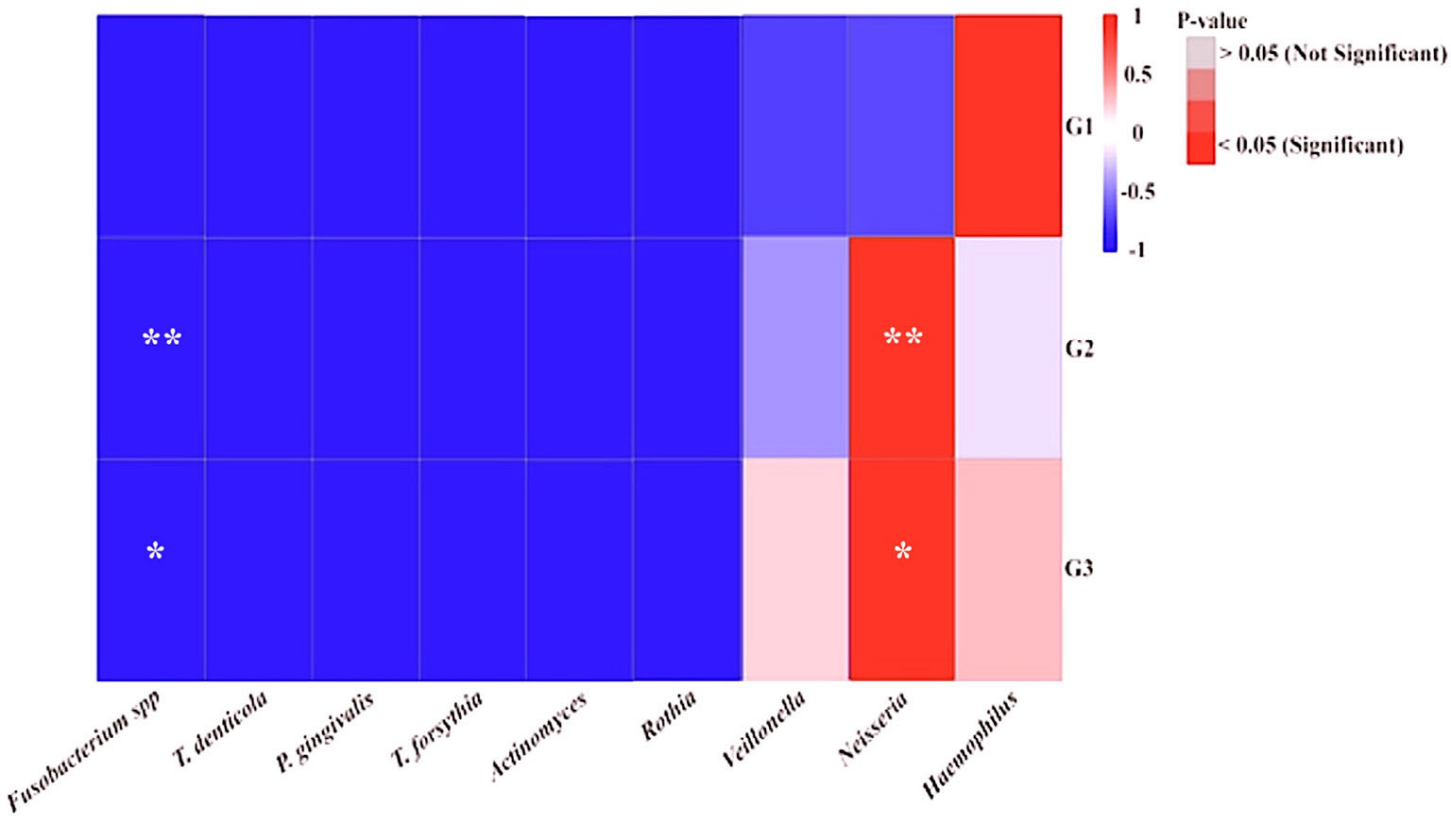
Phylogenetic variations among nitrate-reducing bacteria between T2DM patients with periodontitis (G3) and gingivitis (G2). Overall phylogenetic diversity of nitrate-reducing bacteria was considerably lower in T2DM individuals with periodontitis than in those with gingivitis. Patients with periodontitis (A and B) or gingivitis (C and D), but not both, have specific *Neisseria* species (Blue box). The distribution of *Rothia* species (Red box) also differed among the groupings.

**Figure 4.**
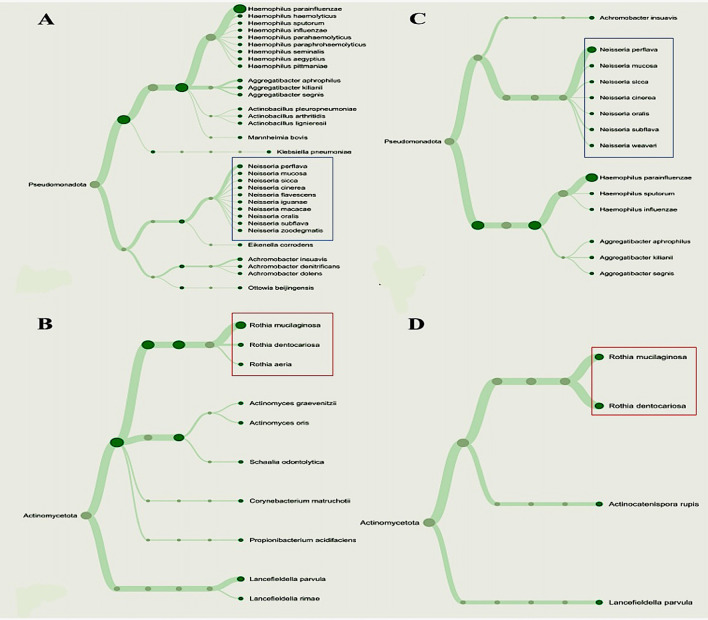
The heatmap displays the quantity of periodontopathic and nitrate-reducing bacteria in the groups under study. The study groups and the targeted bacteria were represented by rows and columns, respectively. The relative proportion of the bacterial assignment within each group is represented by the colors in the heatmap. A shift in color toward dark red denotes a higher abundance. Significant correlations after p-value adjustment are market by *p = 0.05, and **p = 0.01.

**Figure 5.**
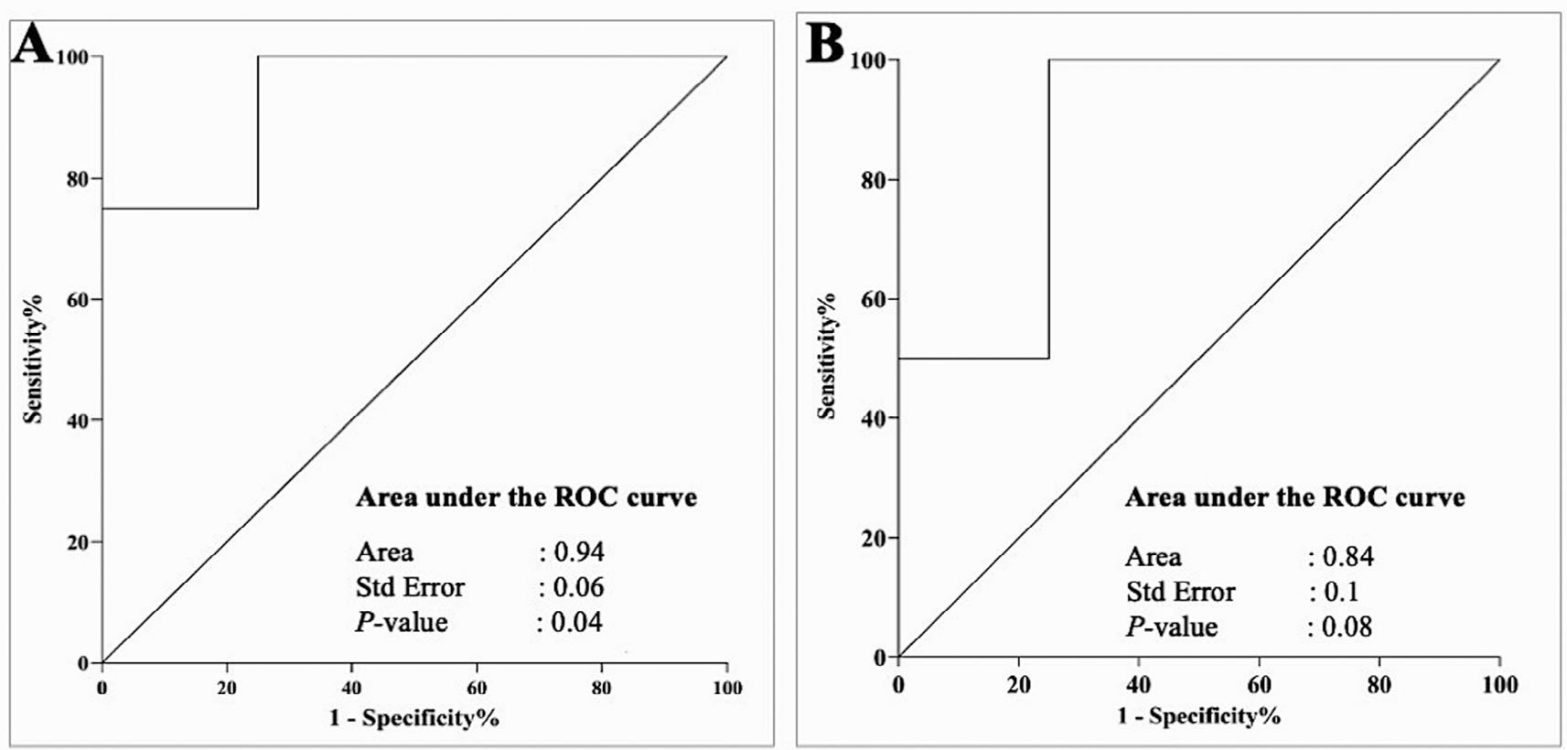
A ROC curve illustrates the relationship between periodontopathic and denitrification bacteria. The interaction between *Fusobacteria* spp. and *Neisseria* is a useful indicator for differentiating between gingivitis and periodontitis, as evidenced by the high AUC (Area Under the Curve) of 0.94 and p-value of 0.04 (A). However, while still a reasonable diagnostic marker (AUC 0.8, p = 0.08), the association between *P. gingivalis* and *Rothia* is not as strong for a prediction (B).

Furthermore, the correlation between specific NRB taxa and periodontopathic bacteria found in the salivary microbiomes of the participants was evaluated using a heat map ( Figure 3). It was shown that each group of diabetic patients had their own unique salivary bacterial profiles. The results also demonstrated that there was a significant positive correlation between the abundance and presence of all periodontopathic bacteria and *Haemophilus* in the group G1, but negative correlations with *Actinomyces* and *Veillonella.* In the G2 and G3 groups of periodontal disorders, *Neisseria* and each periodontopathogen showed a positive association. However, *Fusobacteria* was the only periodontopathogen species in those groups with a strong and moderate interaction. In contrast, *Rothia* was found to be negatively correlated with each periodontopathogen. Given the distinct patterns of relationships, we considered to performing phylogenetic analysis for analyzing the genera *Neisseria* and *Rothia.* We noticed that, of the two NRB species, the gingivitis (G2) group had more variation than the periodontitis (G3) group ( Figure 4). To further contextualize the impacts of the periodontopathogen/NRP relationship, we evaluated their association using a ROC curve, and the findings are illustrated in Figure 5A,B. The correlations that existed between *Fusobacterium* and *Neisseria* and between *P. gingivalis* and *Rothia* had respective area under the curves (AUCs) of 0.93 (p-value = 0.04) and 0.8 (p-value = 0.08).

### Nitrate-Nitrite measurement in saliva

**Figure 6.**
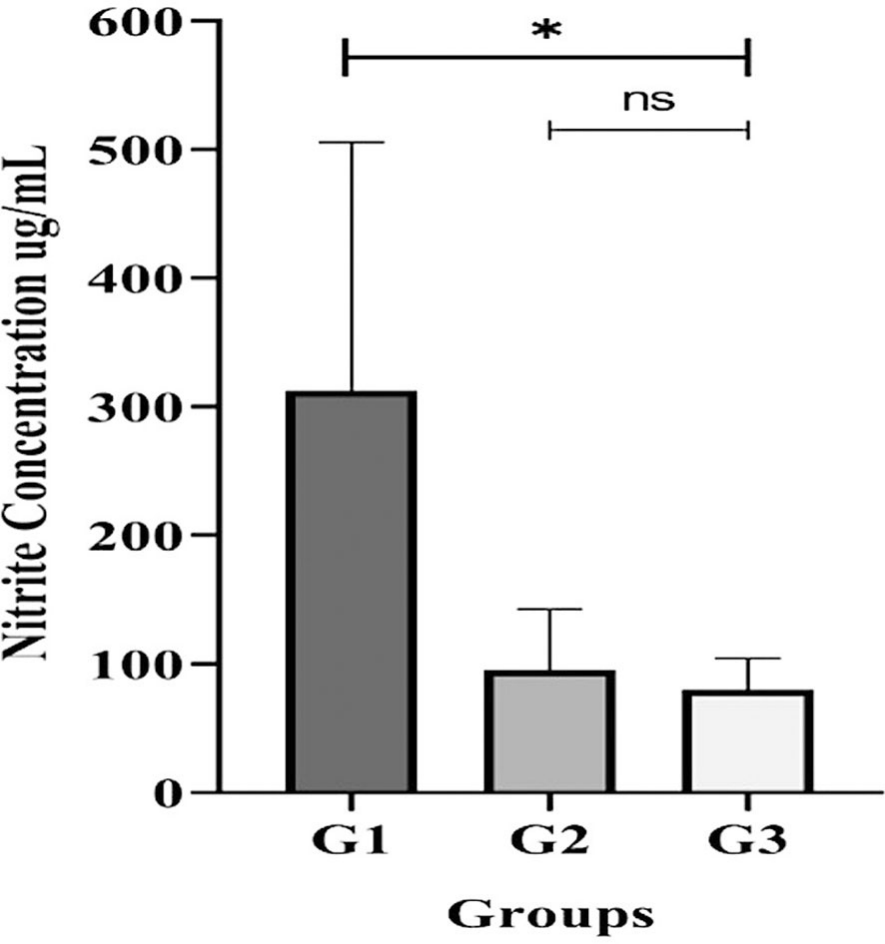
Salivary nitrite concentration in unstimulated saliva from T2DM subject groups. All participants with gingivitis (G2), periodontitis (G3), and no periodontal disease (G1) had their salivary nitrite levels measured using the Griess reaction merthod. Bars represent mean + SD. An asterisk (*) indicates a statistically significant difference (p-value < 0.05) and ns = not significant.

Participants with T2DM accompany with gingivitis or periodontitis (G2 or G3 groups) had significantly lower salivary nitrite concentrations than those without periodontal disease (G1 group) ( Figure 6).

## Discussion

This study’s main finding is that diabetes mellitus and periodontal diseases together have significantly greater effects on alterations in the composition of salivary microbiota than do diabetes mellitus alone. This suggests that the most important factors modifying the composition of salivary microorganisms are periodontal diseases (gingivitis and periodontitis). Thus, we evaluated which oral bacteria increase the risk of periodontal diseases and how specifically diabetes affects them from the perspective of the oral microbiome. Since both polymicrobial synergy and dysbiosis have a major influence on periodontal disorders, ^32, 33^ we intended to better understand any potential relationships between the relative abundance of periodontopathogens and nitrate-reducing bacteria, in diabetics with and without periodontal diseases.

First, we found that the 16S rRNA-based MinION technology’s sequencing depth allowed for the identification of 97% of the bacterial population, allowing the ability to identify bacterial cells in pooled saliva samples. Subsequently, these studies’ findings showed that T2DM patients with periodontal diseases had significantly greater alpha diversity in their saliva than those without the disease. The result suggests that the salivary microbiota’s composition significantly shifted from symbiosis to dysbiosis as our subjects’ periodontal health deteriorated. The findings may also imply that the diversity of individual microbial patterns among our diabetes group members may have led to the difference in alpha diversity seen in this study. Additionally, using the Shannon and Simpson indices, we found that T2DM participants with gingivitis (G2 group) and periodontitis (G3 group) had higher species variety than those without periodontal diseases (G1 group). However, people with T2DM and gingivitis may have more low-abundance bacterial species in their saliva, which could explain the higher Shannon index. Although they have little effect on the Simpson’s index, these rare species add to the total diversity measured by the Shannon index. This implies that, despite the fact that gingivitis and periodontitis displayed different clinical symptoms, species abundance rather than species diversity is the primary factor influencing the differences in salivary microbiome between the two groups. Further research is necessary to fully understand the implications of these findings and their potential therapeutic relevance. However, it should be remembered that these diversity shifts may not always be associated with changes in the relative abundances of the microbiome; they may instead be explained by certain ecological conditions that influence the patterns of microbial succession. ^34^

These results allowed us to distinguish between the two distinct “microbiota states” associated with the G2 and G3 groups of gingivitis and periodontitis, respectively. At the phylum level, *Bacillota* ( *Firmicutes*) are consistently the most prevalent bacteria across all groups, but their relative abundance in the G2 and G3 groups appears to be slightly higher than in the G1 group (those without periodontal disease). Shannon’s diversity index additionally indicated that the changes were most noticeable at the genus level, with *Streptococcus* being the most prevalent bacteria found in the current study. The results were similar to those published by Shaalan et al. (2022) ^35^ and Omori et al. (2022). ^36^ Since the phylum *Bacillota*, which includes numerous genera, is involved in both periodontal health and diseases, ^37, 38^ higher proportions of patients with type 2 diabetes who have periodontal disease may indicate a dysbiosis, in which bacteria from this phylum predominate and exacerbate the inflammatory process of periodontal disease.

Additionally, we discovered that the gingivitis (G1 group) and periodontitis G2 group) have a greater proportion of *Pseudomonadota.* This phyla is the second largest group in the oral microbiome, ^39^ and oral pathobionts may belong to this phylum. ^40^ By comparing to *Bacteroidota*, *Pseudomonadota* was considerably more common in patients with gingivitis (p< 0.0001) but less prevalent in patients with periodontitis. A similar finding was also reported in China, where younger patients with T2DM had a significant abundance of *Proteobacteria* (synonym *Pseuodomonadota*). ^41^ Even though our adult diabetic participants may be more susceptible to early inflammation of periodontal disease due to *Pseudomonadota*-related bacteria, the relative abundance of *Actinomycetota* did not differ significantly across all groups assessed, which is consistent with previous reports. ^42^

One possible explanation for the bacterial shifting mentioned above is the decrease in number or extinction of bacterial species related to nitrate reducers. Therefore, we aimed to determine if the observed compositionality of microbiota in each group could be explained by different prevalence rates of nitrate reducer bacteria. We noticed, that several genera, including *Porphyromonas*, *Fusobacterium*, *Haemophylus*, *Neisseri*a, *Rothia*, and *Veillonela*, were found in this study. All of them have the ability to regulate nitrate-nitrite-nitric oxide (NO). ^16, 43– 45^ Among these bacteria, *Porphyromonas* and *Fusobacterium* are periodontopathic bacteria, ^46^ while the remaining bacteria are linked to oral health. ^47, 48^ This study found that among T2DM patients with periodontal diseases (G2 and G3 groups), *Neisseria* and periodontopathic bacteria ( *P. gingivalis* and *Fusobacteria* spp.) had a significant positive correlation (p< 0.005), as compared with those without periodontal diseases (G1 group). Furthermore, the existence of *Rothia* species in both groups raises the possibility that the main differences or functional role of the species may change depending on the severity of periodontal disease and type 2 diabetes. On the other hand, this highlights *Neisseria*’s significance as a key element of the oral cavity’s core microbiota, ^49^ and this studies demonstrated how the nitrate reducer bacteria and periodontopathogen collaborate to boost periodontal inflammation in individuals with type 2 diabetes. ^50^ Indeed, finding that people with type 2 diabetes who also have periodontal disease have a low amount of *Rothia* in their saliva indicates that the bacteria could benefit people with periodontal health. ^45, 51, 52^ Therefore, in our diabetic patients, *Neisseria* spp. and *Rothia* spp. could be the main oral nitrate-reducing bacteria ^51^ in responsible for periodontal health and inflammation. Consequently, to simplify the interpretation and presentation of our results, we disregarded the other nitrate-reducing bacteria that did not demonstrate a significant association. As a result, we discovered that T2DM patients with periodontitis had much less phylogenetic diversity of these bacteria than those with gingivitis. We noticed, *Neisseria flavescens*, *N. iguanae*, *N. macacae*, *N. oralis* and *N. zoodegmatis* were only found in the pooled metagenomic salivary samples of the gingivitis patient (G2 group), while *N. weaveri* was detected only in the periodontitis patients (G3). The zoonotic pathogens *Neisseria zoodegmatis* and *N. weaveri* have been reported to cause soft tissue infections, ^53, 54^even though their presence in the human salivary microbiome has not been reported.

For *Rothia*, *R. aeria* was exclusively present in the G2 group, while *R. mucilaginosa* and *R. dentocariosa* were discovered in both patient groups (G2 and G3). Indeed, we speculated that the way that bacteria interact with periodontopathogen in T2DM patients may be indicative that gingivitis develops into periodontitis. In this sense, the differences between *Neisseria* or *Rothia* association with the specific bacteria that are commonly associated with periodontal disease can be used to predict the progress of periodontal disease in patients with type 2 diabetes. First, we focused on the red complex bacteria ( *P. gingivalis*, *T. denticola*, and *T. forsythia*), and *Fusobacterium* spp. which are the most threatening or potential pathogens that contribute to periodontal disease in adults. ^55^ We noted, that the red complex bacteria were found in the saliva of each participant group, supporting previous findings that periodontal pathogens were present in individuals with both periodontal disease and periodontal health,. ^56, 57^ Accordingly, group G3 (diabetic individuals with periodontitis) had the highest abundance of periodontopathogens, especially *P. gingivalis.* This finding suggests a more severe dysbiosis in this population, where *P. gingivalis* plays a crucial role. ^9^ The result is in line with a study that examined the subgingival microbiota of individuals with periodontal disease. ^34^ However, it should be mentioned that variations in the host’s specific response to the opportunistic infection may have important variable in the disease’s severity. ^58^ Furthermore, it’s also noteworthy that our diabetes patients have a significantly higher density of bridging periodontopathic bacteria ( *Fusobacterium* spp.), which have been shown to restrict and prepare the environment for the colonization of pathogenic red complex species that lead to periodontitis. ^59, 60^ The high concentration of *Fusobacterium* DNA found in this study suggests that more periodontopathic bacteria colonized the oral cavity of patients with T2DM. In addition, *Fusobacterium* spp. are associated to bleeding on probing, ^61^ a symptom that is more common in our T2DM patients who do not have any periodontal disease (not shown).

Subsequently, our finding which include the dendrogram analysis, indicate a higher diversity of *Neisseria* and *Rothia* species in gingivitis (G2 group) than in periodontitis (G3 group). Our result suggests that specific species within these genera may be more influential in the initiation and early progression of gingival inflammation, while their roles might shift or diminish in chronic periodontitis. ^62^ *Neisseria* may contribute synergistically with periodontopathic bacteria like *Fusobacterium* to establish the inflammation, whereas *Rothia* appears to have a unique, potentially protective, association with the inflammatory process. ^62^

Moreover, it has been reported that the abundance of *Neisseria* in saliva is associated with periodontal health ^63^ and the abundance of *Rothia* decreased in periodontitis. ^13^ Both bacteria are essential for reducing oral nitrate. ^51, 52^ Another study found that while the overall diversity of the oral microbiome declined in mice with diabetes mellitus, *Proteobacteria* along with *Firmicutes* numbers increased. ^64^ The information support our findings that elevated *Proteobacteria* abundance, with *Neisseria* being the most prevalent, seemed to be triggered by inflammation of the periodontal tissue in people with type 2 diabetes. However, we recommend more studies to validate these findings.

Since *Actinomycetota*’s *Rothia* are known NO ^-^_3_ reducers, ^65^ the lower salivary nitric oxide content indicated by the Griess reaction could be the reason why our T2DM patients with periodontal disease (G2 and G3 groups) had a smaller number of these bacteria than those without periodontal disease (G1 group). Nonetheless, the nitrate concentration of the G2 and G3 groups were comparable. This suggests that the advancement of periodontal disease (gingivitis and periodontitis) may impair the activity of nitrate reducer in either periodontal inflammation conditions. ^12^ Interestingly, groups G2 and G3 displayed a decrease in salivary nitrate concentration despite a higher number of *Neisseria.* Hence, this study indicate that there is a complex interaction between different bacterial species and their metabolic activity in the oral microbiome of our T2DM subjects, which is not covered in our study. Indeed, additional study is required to ascertain whether the increased *Neisseria* proportion in the microbiome of T2DM subjects with gingivitis or periodontitis causes or results from periodontal inflammation.

It should be mentioned that the significant variation in bacterial counts between the gingivitis and periodontitis groups in our T2DM participants is not necessarily indicative of a biomarker’s diagnostic value. ^66^ Hence, the capacity to distinguish between cases of gingivitis and periodontitis in individuals with type 2 diabetes was enhanced by the use of the ROC curve, which showed a cut-off point that established a sensitivity and specificity relationship. According to the ROC analysis, the association between *Fusobacteria* and *Neisseria* was a more reliable predictor of gingivitis than the association between *P. gingivalis* and *Rothia* for periodontitis prediction. A stronger AUC (0.93 vs. 0.8) and a lower p-value (0.04 vs. 0.08) indicate a more robust and statistically significant association.

Literature shows, that periodontopathic bacteria ( *P. gingivalis*, *T. denticola*, *T. forsythia*, and *Fusobacterium* spp.) are responsible for the development and progress of periodontal disorders. ^67^ The study’s findings clearly showed that people with type 2 diabetes accompanied with gingivitis or periodontitis have reduced nitrate reduction efficiency, with the exception of *Neisseria*, which affects the ability of microorganisms to reduce nitrate in saliva. ^12^ Therefore, the shifting of oral microbial equilibrium, particularly the balance of nitrate-reducing bacteria activities, may be the cause of periodontal inflammation in our diabetic subjects. Furthermore, a previous study ^68^ demonstrated that the activity of bacteria that reduce nitrate may help minimize the risk of systemic diseases like hypertension and insulin resistance. Our research showed that these bacteria induce gingivitis prior to generate periodontitis. Nevertheless, more study is required to validate this finding.

### Limitation

First, the cross-sectional approach to the study renders it difficult to determine causal relationships. Second, the pooled metagenomes are not representative enough to reveal any significant differences between the groups being studied. ^69^ Nonetheless, the findings confirm and reinforce the results of the 16S rRNA gene sequencing analysis. Lastly, we did not include smoking habit, which may be a confounder in the study results. However, data are emerging that the oral microbiota, which is associated with periodontal disease, may be strongly correlated with the incidence of type 2 diabetes, even when confounders are excluded.

## Conclusion

The results of the study revealed that the microbial communities in saliva change significantly between the conditions of oral health and periodontal disease, with the progression of periodontal inflammation in diabetics being an important contributor in these variations. Overall, the findings demonstrate that individuals with type 2 diabetes mellitus who also have gingivitis or periodontitis may have unique relationships between periodontopathic and nitrate-reducing bacteria in their salivary microbiome. These characteristic could be crucial microbiological indicators for the early detection and management of gingivitis in order to prevent periodontitis.

## Author contributions

BB: Data curation, Funding acquisition, Writing-review & editing. DT: Validation, Visualization, Review & editing. CT: Resources, Supervision, Review &editing. NH:, Resources, Validation. CT: Project administration, Validation.YS: Data curation, Validation. SS: Validation, Visualization, Review & editing. AW: Data curation, Validation. FR: Resources and Supervision FT: Data curation, Validation, Visualization. DA: Resources and Supervision. EB: Conceptualization, Writing-Original draft.

## Data Availability

Figshare: Raw data salivary microbiome using 16s barcoding kit 24 V14, ONT (Oxford Nanopore Technology), https://doi.org/10.6084/m9.figshare.28365782.v4 ^70^ This project contains the following underlying data:
• FASTQ files in folder of barcode 18, 19, 20 • Subject data in.xlsx format •
Figures in PNG and JPG format Data are available under the terms of the Creative Commons Zero “No rights reserved” data waiver (CC0 1.0 Public domain dedication). In accordance with the criteria of the institutional ethics committee, All participants provided written informed consent before they participated part in the study, and the Dr. Cipto Mangunkusumo Hospital’s Ethics Committee approved the study’s protocols (Ethics Reference Number: KET-1203/UN2.F1/ETIK/PPM.06.02/2023).
