# Salivary microbiome and periodontopathogen/denitrifying bacteria associated with gingivitis and periodontitis in people with type 2-diabetes

**DOI:** 10.12688/f1000research.161731.3

**Published:** 2025-12-09

**Authors:** Endang Bachtiar, Boy M. Bachtiar, Dicky L Tahapary, Turmidzi Fath, Citra Fragrantia Theodora, Natalina Haerani, Selvi Nafisa Shahab, Yuniarti Soeroso, Ardy Wildan, Fergie Marie Joe Grizella Runtu, Fatimah Maria Tadjoedin, Dewi Ayuningtyas

**Affiliations:** 1 Oral Biology Fac. of Dentistry, University of Indonesia, Depok, West Java, 16424, Indonesia; 2 Clinical Research Unit RSCM, . Metabolic-Endocrine-Diabetes Division, Dept. Internal Medicine, Universitas Indonesia, Depok, West Java, Indonesia; 3 Faculty of Dentistry, Department of Oral Biology and Oral Science Research Center, Universitas Indonesia, Depok, West Java, 10430, Indonesia; 4 Faculty of Dentistry,Department of Periodontology, Universitas Indonesia, Jakarta, Jakarta, 10430, Indonesia; 5 Faculty of Medicine, Department Microbiology, Clinical Research Unit RSCM, Universitas Indonesia, Ciptomangunkusumo Hospital., West Java, Indonesia; 6 Metabolic-Endocrine-Diabetes Division, Dept. Internal Medicine, Universitas Indonesia, Depok, West Java, Indonesia

**Keywords:** Diabetes; gingivitis; periodontitis; salivary microbiome; nanopore sequencing

## Abstract

**Background:**

Despite diabetes mellitus and periodontal diseases are mutually exclusive, little is known about particular types of bacteria that may have exacerbated the development of diabetics’ periodontal inflammation. The purpose of this study was to descriptively characterize and explore the differences in the salivary microbiomes of individuals with type 2 diabetes (20-40 years old) who had gingivitis or periodontitis to those who did not. Additionally, we evaluated the descriptive relationship between the relative abundance of periodontopathogens and nitrate-reducing bacteria in their salivary microbiome.

**Methods:**

Saliva was collected from all participants. Genomic DNA was isolated and pooled in equimolar quantities from all individuals within each group to create three pooled libraries: type 2 diabetes (T2DM) patients without periodontal disease (G1), T2DM patients with gingivitis (G2), and T2DM patients with periodontitis (G3). Sequencing was performed using Oxford Nanopore MinION Technology. The relative abundance and bacterial diversity were measured using bioinformatic methods, and all analyses of sequencing data were strictly descriptive and exploratory. Salivary nitrite/nitrate concentrations were measured on individual, un-pooled samples.

**Results:**

The salivary microbiota among people with type 2 diabetes and periodontal disease (G2; G3) was observed to have greater bacterial diversity and abundance than that of patients without periodontal disease (G1), according to descriptive alpha-diversity analysis. The G3 group exhibited the largest relative abundance of *Porphyromonas gingivalis*, a key periodontopathogen. Descriptive analysis also suggested that periodontopathic bacteria and nitrate-reducing bacteria have different community structures across the groups. Furthermore, comparison of individual salivary samples showed that nitrite/nitrate concentration was significantly lower in the G3 group compared to the G1 group (p< 0.05).

**Conclusion:**

Results of this exploratory study suggest that the relationship between periodontopathic and denitrifying bacteria in the salivary microbiome varies among those with type 2 diabetes mellitus who also have gingivitis or periodontitis. These distinct microbial features observed may be microbiological characteristics associated with the progression of periodontal disease in this population, warranting further validation as potential indicators for early management.

## Introduction

The development of periodontal disease is known to be significantly influenced by the oral microbiota, which may also be an important variable in systemic disorders including diabetes and heart disease. ^1^ Additionally, recent studies indicate that those with type 2 diabetes (T2DM) are more likely to develop periodontitis and to have more severe forms of disease. ^2– 4^ Even though numerous studies have looked at the bidirectional relationship between diabetes mellitus and periodontal disorders, there are still gaps in the quantity and quality of study on the subject. ^5, 6^ For example, existing literature has explored the link between diabetes and periodontal disease, but these studies often have limitations. While some have been constrained by comparatively small sample sizes, ^7, 8^ others have concentrated on particular geographic regions. ^9, 10^ Furthermore, although current literature ^1, 2^ indicates that therapeutic medications like chlorhexidine are commonly used to manage oral bacteria and that microRNAs (miRNAs) are significant regulators of inflammation, studies are still ongoing to determine the precise microbial changes and how they relate to host factors. Our study expands on that approach by specifically looking at the salivary microbiome to identify potential microbial indicators for disease progression.

The most common types of periodontal disease are gingivitis and periodontitis. In many respects, gingivitis is a state of stable inflammation that represents equilibrium (homeostasis). Hence, gingivitis is mostly reversible. ^11^ However, the prolonged or recurrent occurrence of the condition, particularly in predisposed individuals, may lead to irreversible destruction of hard and soft tissues (periodontitis). Both gingivitis and periodontitis have been recognized as polymicrobial, biofilm-based inflammatory diseases ^12^ in which a disruption of the ecological balance between the biofilm and the periodontal tissue homeostasis plays a more important role than specific infections, ^13– 15^ alongside other environmental, lifestyle and genetic risk factors.

In patients with periodontitis, the number of periodontopathogens ( *Porphyromonas gingivalis*, *Treponema denticola*, and *Tannerella forsythia*) and *Fusobacterium* spp. is increasing, while the number of normal flora bacteria is decreasing. ^16– 18^ Healthy microbial populations include all genera; *Rothia, Neisseria, Actinomyces, Veillonella, Kingella*, *Propionibacterium*, *Prevotella, Granulicatella,* and *Haemophilus.* They are all recognized to be nitrate-reducing bacteria (NRB), ^19, 20^ and have antimetabolic disease activities. ^21^ These NRB have attracted much attention currently given their crucial function in the human nitrogen cycle’s nitrate (NO3-) - nitrite (NO2-) - nitric oxide (NO) pathway. ^22, 23^ Additionally, blood pressure regulation and insulin resistance are positively impacted by oral nitrate-reducing bacteria. ^24^ The intricate relationship between nitrate-reducing bacteria (NRB) and periodontopathogens has not been well investigated in many studies. Our previous study, which used real-time PCR, ^25^ showed that individuals with T2DM who did not have periodontitis had different prevalence of specific periodontopathogens and nitrate-reducing bacteria. Therefore, the purpose of this study was to descriptively characterize and explore the community structure and differences in the salivary microbiomes of individuals with T2DM who had gingivitis or periodontitis to those who did not. We focused specifically on descriptive interplay between key periodontopathogens (such as *P. gingivalis* and *Fusobacterium spp*.) and nitrate-reducing bacteria (NRB) community (including *Haemophilus*, *Neisseria, Rothia*, and *Veillonella*). We hypothesize that the unique metabolic environment of diabetic patients facilitates disease progression via a functional shift – a disruption in the beneficial nitric oxide (NO)-production function of the NRB community – which is associated with increased abundance of periodontopathic species.

In addition to subgingival biofilm (SGB), saliva was chosen as the sample type to accurately capture the oral microbiome’s composition, since it is commonly used to explain the microbial shifts that occur in periodontal sites as they progress from health to disease. ^26– 29^ Furthermore, the Oxford Nanopore Technology (ONT) long-read sequencing approach was performed to identify taxa based on whole 16S rRNA sequences, which allows the species-level identification of the representative microbes, including periodontopathogen ^30^ and oral bacteria associated with nitrate-reducing. ^31^

## Methods

### Participants and patient characteristic

Participants in this cross-sectional study were Indonesian adult patients recruited from the Dr. Cipto Mangunkusumo Hospital in Jakarta between November 2023 and January 2024. Participants were selected to have at least 20 teeth without any obvious evidence of root or oral mucosal caries, be within the ages of 20 and 40, and have a diagnosis of non-insulin-dependent Type 2 Diabetes Mellitus diagnosed by the internist of the Division of Endocrinology, based on the conditions of blood glucose level 2 hours after oral glucose load ≥ 200 mg/dL, HbA1c ≥ 6.5%, or plasma glucose was ≥ 200 mg/dl with classic hyperglycemic crisis (not shown). In addition, participants had not smoked within three months, were not taking antibiotics or nonsteroidal anti-inflammatory medications, and had not had periodontal surgery or therapy within the preceding six months. Among the exclusion criteria were being pregnant or nursing, using specific drugs (such hormones) within six months of sample collection, or having a metabolic disorder other than diabetes. Two competent and experienced periodontists assessed each participant’s periodontal health. According to current classification guidelines, the diagnosis was only made based on clinical features like Clinical Attachment (CAL>2 mm) and Probing Depth (PD>4 mm). We recognize that our review did not take radiographic evidence of alveolar bone loss into account. The examiner was calibrated before starting the investigation to guarantee measurement reliability and consistency. The inter-examiner reliability was evaluated using the Kappa test, with a value of 0.86.

The diagnosis of gingivitis was made by bleeding on probing (BOP) score, ^32^ while chronic periodontitis was diagnosed based on the standard classification of the America academic of periodontology, ^33^ without radiological evaluation to support the use of the North Caroline periodontal probe (UNC-15). Participants diagnosed with chronic periodontitis were identified as having at least 30% of sites with alveolar bone resorption, as well as more than 4 sites with probing depth (PD) ≥ 4 mm and clinical attachment loss (CAL) ≥ 2 mm.

In accordance with the criteria of the institutional ethics committee, all participants provided written informed consent before they participated part in the study, and the Dr. Cipto Mangunkusumo Hospital’s Ethics Committee approved the study’s protocols (Ethics Reference Number: KET-1203/UN2.F1/ETIK/PPM.00.02/2023).

Data availability note: Clinical data on Body Mass Index (BMI), HBA1c levels, and duration of T2DM were not available for inclusion in this manuscript due to institutional privacy and ethical agreements with the collaborating Faculty of Medicine Universitas Indonesia.

### Saliva sample collection, DNA extraction, and sequencing

Following a protocol described elsewhere, ^34^ after rinsing their mouths, using 0.9% normal saline for about 30s, approximately 3-5 mL of unstimulated whole saliva was collected from participants, one hour after they ceased eating, drinking, or brushing their teeth, and prior to any clinical periodontal assessment to prevent potential interference from bleeding or mechanical irritation. For DNA analysis, 1.5 mL of the sample was immediately treated with DNA stabilization buffer, and genomic DNA was isolated. The remaining saliva was immediately aliquoted for chemical analysis and stored at -80°C until analysis. These aliquoted saliva samples were maintained individually and were not pooled.

DNA was extracted using the Monarch ^®^^,^™ Genomic DNA purification kit, NEB #T3010S/L (New England Biolabs, Bruningstrasse Frankfurt am Main, Germany) and quantified using a Qubit 2.0 Fluorometer (Invitrogen, Carlsbag, CA, USA). The inclusion of negative controls, or no-template controls, in the DNA extraction and PCR amplification processes allowed us to verify the validity of our findings by looking for contamination. To further validate the effectiveness of the PCR reaction, a positive control that came with the 16S Barcoding Kit was utilized.

DNA pooling and sequencing: Furthermore, the genomic DNA of each study group’s samples was ligated in equimolar quantities to create a pool of three group libraries for nanopore sequencing. Each barcoding kit contained 50 ng of starting DNA. Nanopore amplicon library was prepared using the 16S Barcoding Kit 24 V14 (SQK-RAB204, Oxford Nanopore Technologies, UK) following the manufacturer’s instruction. Primers 27F and 1492R are included in the kit to amplify the whole 16S rRNA gene.

Sequencing was conducted using the MinION (Oxford Nanopore Technologies, UK) with a MinION flow cell (R10.4.1) for 8 hours. Subsequently, the basecalling were generated using MinKNOW (Oxford Nanopore Technologies, UK). For microbiota profiling analysis, we followed the EPI2ME for wf-metagenomic workflow for real time analysis. The analysis results were further generated in the form of a report in the EPI2ME for wf-metagenomic.

Filtering was implemented before the generation of the relative abundance table. Raw sequencing data were base-called utilising Dorado (v7.6.8), and reads were trimmed and filtered according to a minimal quality score (Q-score ≥ 8). The resulting pass reads were further processed with the Nextflow wf-metagenomics pipeline, and only reads within the length range of 200–1500 bp were included for taxonomic classification using Kraken2 (ncbi_16s_18s_28s_ITS database). As our focus was on reporting the relative abundances derived from high-quality filtered reads. Only the several abundant genera or species (periodontopathogen/denitrifying bacteria associated with gingivitis and periodontitis) were displayed, while the others were grouped as ‘Others’.

### Bioinformatic preprocessing and descriptive analysis (pooled DNA)

For microbiota profiling analysis (OTU, alpha diversity, and rarefaction curve), the EPI2ME for wf-metagenomic was used. To ensure data quality, only high-quality reads (“pass”, >5 cumulative reads) were included in the analysis. ^35^ Raw sequencing data were base-called utilising Dorado (v7.6.8), and reads were trimmed and filtered according to a minimal quality score (Q-score ≥8). The resulting pass read were further process with the netflow wf-metagenomics pipeline, and taxonomic classification using Kraken2 (ncbi_16s_18s_28s_ITS database). The operational taxonomic units (OTUs) in each group and the rarefaction curve were developed using EPI2ME, and analysed using RStudio 4.3.2.

The analysis of the pooled sequencing data, including alpha diversity (Simpson and Shannon indices) and taxonomic comparisons, was strictly descriptive and exploratory. No inferential statistic (such as Kruskal-Wallis test, t-test, or one way ANOVA) were applied to the sequencing data, as the pooling of samples precludes the estimation of inter-individual variance. Only the several abundant genera or targeted species were displayed, while others were grouped as “Others”.

### Salivary nitrite/nitrate measurement and inferential analysis

Aliquoted individual saliva samples were thawed, and the total salivary nitrite/nitrate concentrations were quantified for each participant using Griess Reagent System (Promega #TB229, Madison, WI 53711-5399 USA), ^24^ allowing the mixture remain at room temperature for 10 minutes in the dark, and then using a spectrophotometer (AccuReader. M965/M965+, Nangang, Taipei, Taiwan), to quantify the mixture at 450 nm.

For salivary nitrite/nitrate levels, which were measured on individual participants, descriptive statistics are presented as Mean **±** Standard Deviation (SD). Differences in mean values were determined by one-way ANOVA test or Kruskal-Wallis test. GraphPad Prism software version 10 was used to perform these analyses. A significance level of p< 0.05 was employed for all inferential tests performed on the individual-level data.

## Results

**Table 1.** Baseline demographic and clinical characteristics of study. Characteristics are shown as Mean ± Standard Deviation (SD) for continuous variables and as absolute number (n) and percentage (%) for categorical variables (Sex). Differences between the three study groups were assessed using one-way ANOVA for parametric data (Age) and the Kruskal-Wallis test for non-parametric variables (PD, CAL, % Sites with BOP). Group 1 (G1) = No periodontal disease, Group 2 (G2) = Gingivitis, and Group 3 (G3) = periodontal disease.

Characteristic	No Periodontal disease (G1)	Gingivitis (G2)	Periodontitis (G3)	p-value
**Total participants, n (%)**	n = 29 (17%)	n = 54 (31%)	n = 89 (52%)	-
**Age (years), Mean ± SD**	40.94 ± 7.64	43.3 ± 10.3	50.07 ± 8.84	p > 0.05
Male, n (%)	16 (55.2%)	24 (44.4%)	40 (44.9%)	p < 0.05
Female, n (%)	13 (44.8%)	30 (55.6%)	49 (55.1%)	p < 0.05
**Probing depth (PD, mm)** **Mean ± SD**	2.4 ± 0.1	2.6 **±** 0.3	4.8 **±** 0.2	p < 0.05
**Clinical attachment loss** **(CAL, mm), Mean ± SD**	0.14 ± 0.1	0.2 ± 0.1	2.2 ± 0.2	p < 0.05
**% Sites with BOP** **Mean ± SD**	-	9.7 ± 1.4	10 ± 1.3	p < 0.05

**Figure 1.**
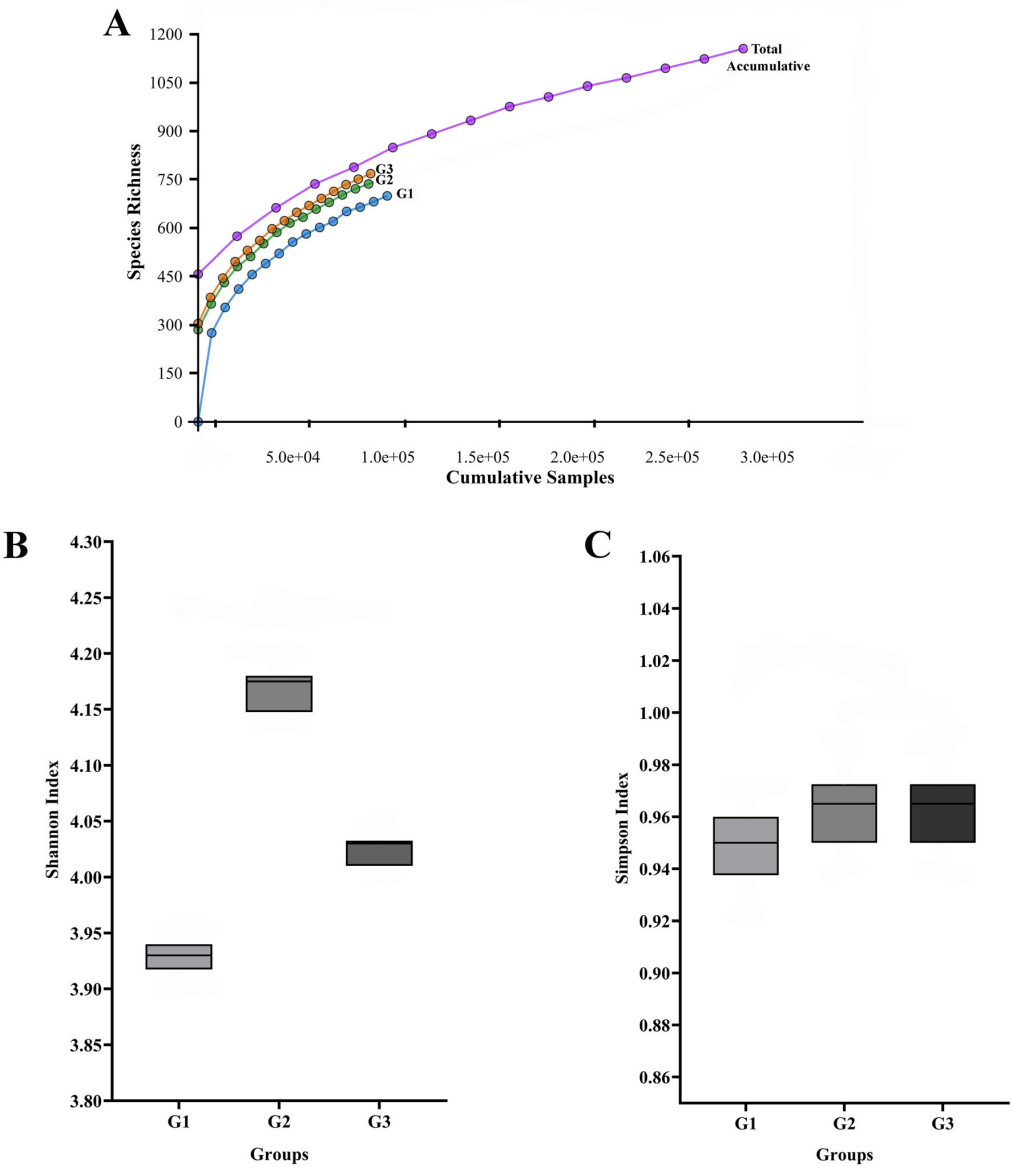
The salivary microbiome's rarefaction curve and alpha diversity among different T2DM patient groups. The total number of sequences is displayed on the horizontal axis, while the number of operational taxonomic units (OTUs) at a 97% intersequence similarity level is shown on the vertical axis of the rarefaction curve (A). The diversity indices of Shannon (B) and Simpson (C) show how alpha diversity varies among the three groups in terms of evenness and richness. Species diversity and evenness seem to be larger in gingivitis group (G2) and periodontitis group (G3) compared to oral health group (G1).

**Figure 2.**
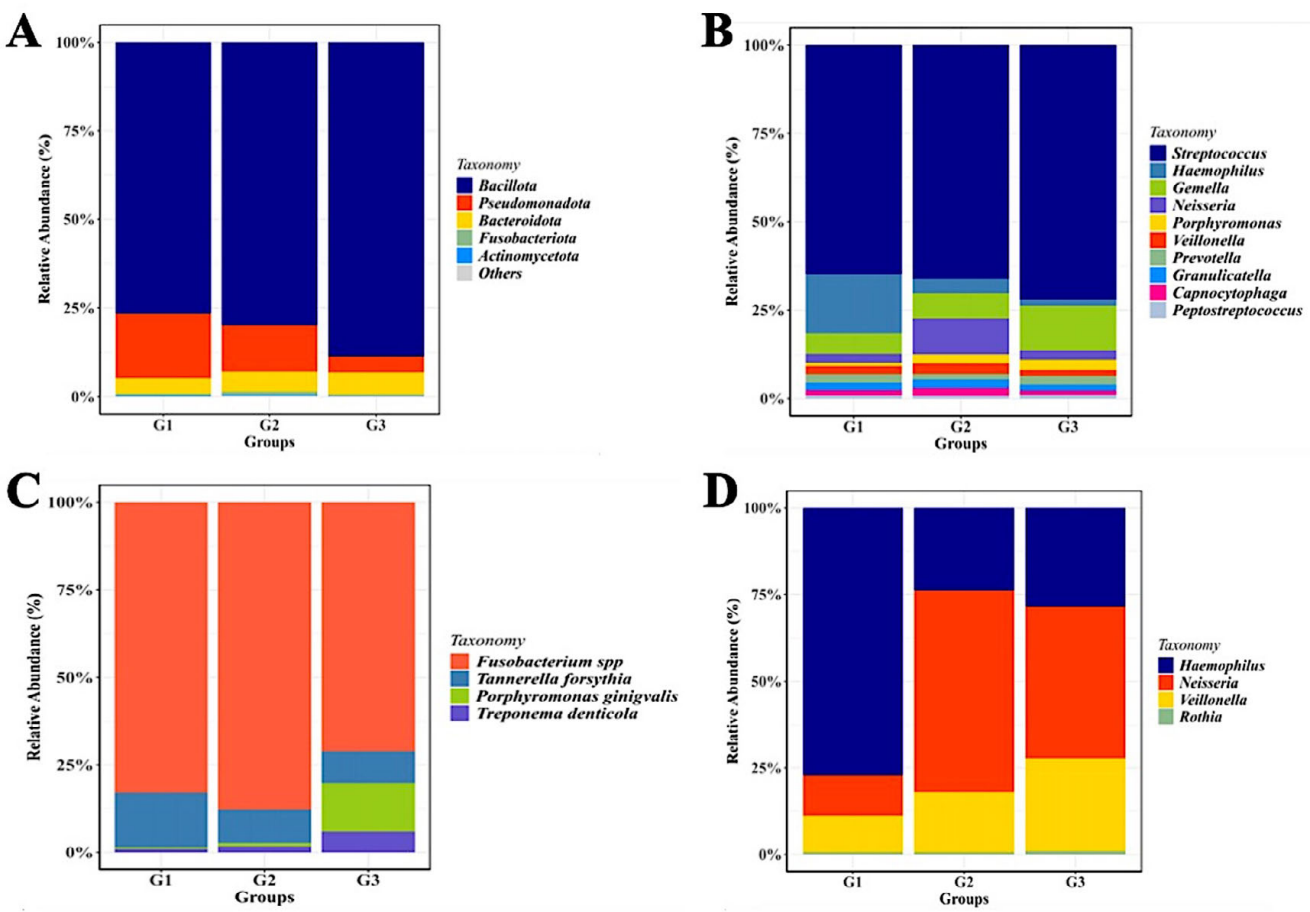
Saliva microbiota taxonomic composition in T2DM patients with and without periodontal diseases. Relative abundance of mayor of salivary bacterial taxa, which relative abundance >5%, are presented at the phylum (A) and genus (B) level. “Others” refers to the remaining phyla. A comparison of the relative abundance of periodontopathic bacterial species (C) and genus of NO _3_^-^-reducing bacteria (D) between three groups of patient with T2DM. G1 through G3 represent the participant group.

**Figure 3.**
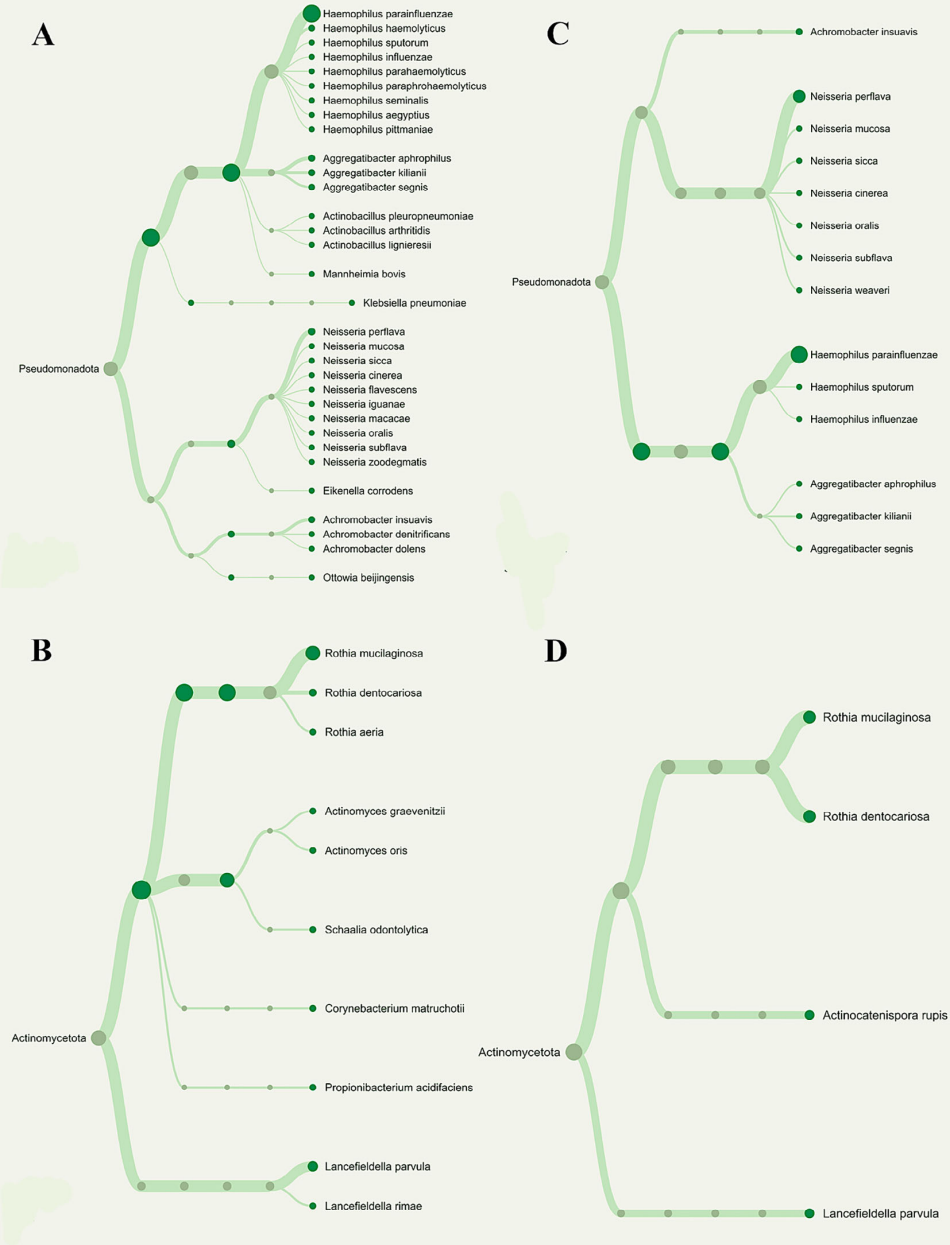
Phylogenetic variations among nitrate-reducing bacteria between T2DM patients with periodontitis (G3) and gingivitis (G2). Overall phylogenetic diversity of nitrate-reducing bacteria was considerably lower in T2DM individuals with periodontitis than in those with gingivitis. Patients with periodontitis (A and B) or gingivitis (C and D), but not both, have specific *Neisseria* species (Blue box). The distribution of *Rothia* species (Red box) also differed among the groupings.

**Figure 4.**
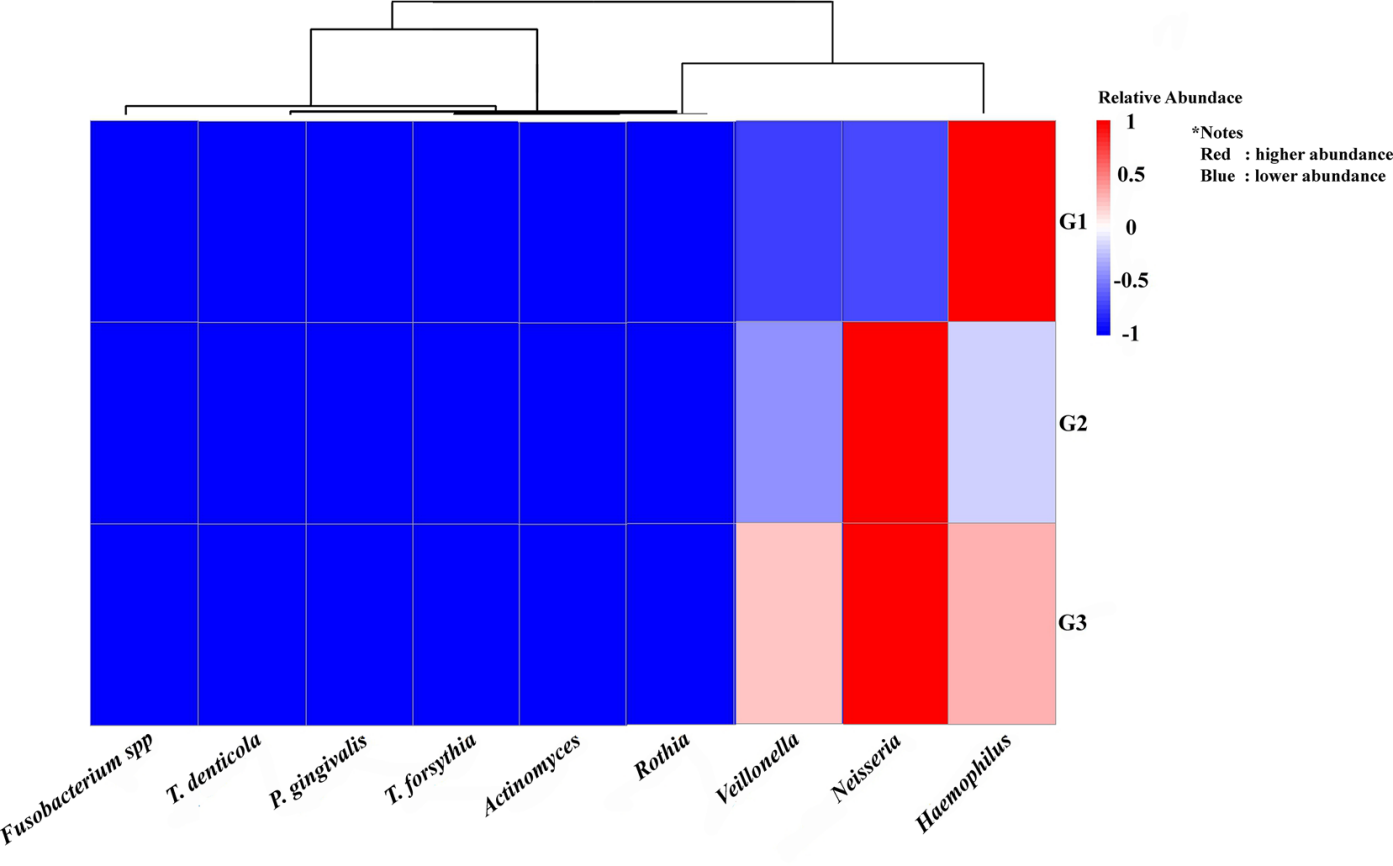
The heatmap displays the quantity of periodontopathic and nitrate-reducing bacteria in the groups under study. The study groups and the targeted bacteria were represented by rows and columns, respectively. The relative proportion of the bacterial assignment within each group is represented by the colors in the heatmap. A shift in color toward dark red denotes a higher abundance.

**Figure 5.**
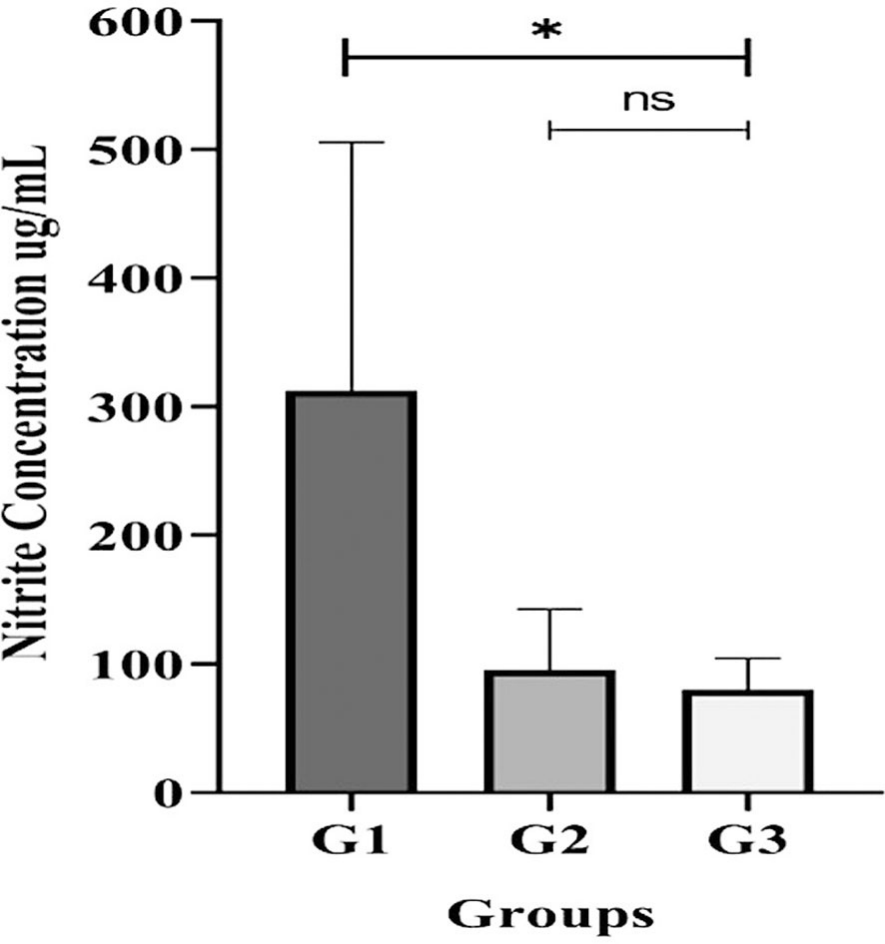
Salivary nitrite concentration in unstimulated saliva from T2DM subject groups. All participants with gingivitis (G2), periodontitis (G3), and no periodontal disease (G1) had their salivary nitrite levels measured using the Griess reaction method. Bars represent mean + SD. An asterisk (*) indicates a statistically significant difference (p-value < 0.05) and ns = not significant.

## Discussion

This study’s main finding is that diabetes mellitus and periodontal diseases together have significantly greater effects on alterations in the composition of salivary microbiota than do diabetes mellitus alone. This descriptively suggests that the most important factors modifying the composition of salivary microorganisms are periodontal diseases (gingivitis and periodontitis). Thus, we evaluated which oral bacteria increase the risk of periodontal diseases and how specifically diabetes affects them from the perspective of the oral microbiome. Since both polymicrobial synergy and dysbiosis have a major influence on periodontal disorders, ^36, 37^ we intended to better understand any potential relationships between the relative abundance of periodontopathogens and nitrate-reducing bacteria, in diabetics with and without periodontal diseases.

First, we found that the 16S rRNA-based MinION technology’s sequencing depth allowed for the identification of 97% of the bacterial population, allowing the ability to identify bacterial cells in pooled saliva samples. Subsequently, these studies’ findings showed that T2DM patients with periodontal diseases were observed to have a greater alpha diversity in their saliva than those without the disease. The result suggests that the salivary microbiota’s composition significantly shifted from symbiosis to dysbiosis as our subjects’ periodontal health deteriorated. The findings may also imply that the diversity of individual microbial patterns among our diabetes group members may have led to the difference in alpha diversity seen in this study. Additionally, using the Shannon and Simpson indices, we descriptively found that T2DM participants with gingivitis (G2 group) and periodontitis (G3 group) had higher species variety than those without periodontal diseases (G1 group). However, people with T2DM and gingivitis may have more low-abundance bacterial species in their saliva, which could explain the higher Shannon index. Although they have little effect on the Simpson’s index, these rare species add to the total diversity measured by the Shannon index. This implies that, despite the fact that gingivitis and periodontitis displayed different clinical symptoms, species abundance rather than species diversity is the primary factor influencing the observed differences in salivary microbiome between the two groups. Further research is necessary to fully understand the implications of these findings and their potential therapeutic relevance. However, it should be remembered that these diversity shifts may not always be associated with changes in the relative abundances of the microbiome; they may instead be explained by certain ecological conditions that influence the patterns of microbial succession. ^38^

These results allowed us to distinguish between the two distinct “microbiota states” associated with the G2 and G3 groups of gingivitis and periodontitis, respectively. At the phylum level, *Bacillota* ( *Firmicutes*) are consistently the most prevalent bacteria across all groups, but their relative abundance in the G2 and G3 groups appears to be slightly higher than in the G1 group (those without periodontal disease). Shannon’s diversity index additionally indicated that the changes were most noticeable at the genus level, with *Streptococcus* being the most prevalent bacteria found in the current study. The results were similar to those published by Shaalan et al. (2022) ^39^ and Omori et al. (2022). ^40^ Since the phylum *Bacillota*, which includes numerous genera, is involved in both periodontal health and diseases, ^41, 42^ higher proportions of patients with type 2 diabetes who have periodontal disease may indicate a dysbiosis, in which bacteria from this phylum predominate and exacerbate the inflammatory process of periodontal disease.

Additionally, we discovered that the gingivitis (G1 group) and periodontitis G2 group) have a greater proportion of *Pseudomonadota.* This phyla is the second largest group in the oral microbiome, ^43^ and oral pathobionts may belong to this phylum. ^44^ Descriptively, *Pseudomonadota* was observed to be more common in patients with gingivitis but less prevalent in patients with periodontitis, relative to *Bacteroidota*. A similar finding was also reported in China, where younger patients with T2DM had a descriptive abundance of *Proteobacteria* (synonym *Pseuodomonadota*). ^9^ Even though our adult diabetic participants may be more susceptible to early inflammation of periodontal disease due to *Pseudomonadota*-related bacteria, the relative abundance of *Actinomycetota* did not differ descriptively across all groups assessed, which is consistent with previous reports. ^45^

One possible explanation for the bacterial shifting mentioned above is the decrease in number or extinction of bacterial species related to nitrate reducers. Therefore, we aimed to determine if the observed compositionality of microbiota in each group could be explained by different prevalence rates of nitrate reducer bacteria. We noticed, that several genera, including *Porphyromonas*, *Fusobacterium*, *Haemophylus*, *Neisseri*a, *Rothia*, and *Veillonela*, were found in this study. All of them have the ability to regulate nitrate-nitrite-nitric oxide (NO). ^20, 46– 48^ Among these bacteria, *Porphyromonas* and *Fusobacterium* are periodontopathic bacteria, ^49^ while the remaining bacteria are linked to oral health. ^50, 51^ Descriptively, this study found that among T2DM patients with periodontal diseases (G2 and G3 groups), the relative abundance of *Neisseria* and periodontopathic bacteria ( *P. gingivalis* and *Fusobacteria* spp.) appeared to follow a similar pattern as compared with those without periodontal diseases (G1 group). Furthermore, the existence of *Rothia* species in both groups raises the possibility that the main differences or functional role of the species may change depending on the severity of periodontal disease and type 2 diabetes. On the other hand, this highlights *Neisseria*’s significance as a key element of the oral cavity’s core microbiota, ^52^ and this descriptive data demonstrated how the nitrate reducer bacteria and periodontopathogen collaborate to boost periodontal inflammation in individuals with type 2 diabetes. ^7^ Indeed, finding that people with type 2 diabetes who also have periodontal disease have a low amount of *Rothia* in their saliva indicates that the bacteria could benefit people with periodontal health. ^48, 53, 54^ Therefore, in our diabetic patients, *Neisseria* spp. and *Rothia* spp. could be the main oral nitrate-reducing bacteria ^53^ in responsible for periodontal health and inflammation. Consequently, to simplify the interpretation and presentation of our results, we disregarded the other nitrate-reducing bacteria that did not demonstrate the largest descriptive difference. As a result, we discovered that T2DM patients with periodontitis had much less phylogenetic diversity of these bacteria than those with gingivitis. We noticed, *Neisseria flavescens*, *N. iguanae*, *N. macacae*, *N. oralis* and *N. zoodegmatis* were only found in the pooled metagenomic salivary samples of the gingivitis patient (G2 group), while *N. weaveri* was detected only in the periodontitis patients (G3). The zoonotic pathogens *Neisseria zoodegmatis* and *N. weaveri* have been reported to cause soft tissue infections, ^55, 56^ even though their presence in the human salivary microbiome has not been reported.

For *Rothia*, *R. aeria* was exclusively present in the G2 group, while *R. mucilaginosa* and *R. dentocariosa* were discovered in both patient groups (G2 and G3). Indeed, we speculated that the way that bacteria interact with periodontopathogen in T2DM patients may be indicative that gingivitis develops into periodontitis. In this sense, the descriptive differences in the association patterns between *Neisseria* or *Rothia* and specific periodontopathic bacteria may be characteristic of the progression of periodontal disease in patients with type 2 diabetes. First, we focused on the red complex bacteria ( *P. gingivalis*, *T. denticola*, and *T. forsythia*), and *Fusobacterium* spp. which are the most threatening or potential pathogens that contribute to periodontal disease in adults. ^57^ We noted, that the red complex bacteria were found in the saliva of each participant group, supporting previous findings that periodontal pathogens were present in individuals with both periodontal disease and periodontal health. ^58, 59^

Accordingly, group G3 (diabetic individuals with periodontitis) had the highest descriptive abundance of periodontopathogens, especially *P. gingivalis.* This finding suggests a more severe dysbiosis in this population, where *P. gingivalis* plays a crucial role. ^13^ The result is in line with a study that examined the subgingival microbiota of individuals with periodontal disease. ^38^ However, it should be mentioned that variations in the host’s specific response to the opportunistic infection may have important variable in the disease’s severity. ^60^ Furthermore, it’s also noteworthy that our diabetes patients have a descriptively higher density of bridging periodontopathic bacteria ( *Fusobacterium* spp.), which have been shown to restrict and prepare the environment for the colonization of pathogenic red complex species that lead to periodontitis. ^61, 62^ The high concentration of *Fusobacterium* DNA found in this study suggests that more periodontopathic bacteria colonized the oral cavity of patients with T2DM. Although a previous study has linked *Fusobacterium* spp. to bleeding on probing (BOP), ^63^ our findings demonstrated that in patient without periodontal disease G1 group), as indicated in Table 1, the absence of gingivitis was consistent with a BOP < 10%. Subsequently, our finding which include the dendrogram analysis, indicate a higher diversity of *Neisseria* and *Rothia* species in gingivitis (G2 group) than in periodontitis (G3 group). Our result suggests that specific species within these genera may be more influential in the initiation and early progression of gingival inflammation, while their roles might shift or diminish in chronic periodontitis. ^64^ *Neisseria* may contribute synergistically with periodontopathic bacteria like *Fusobacterium* to establish the inflammation, whereas *Rothia* appears to have a unique, potentially protective, association with the inflammatory process. ^64^

Moreover, it has been reported that the abundance of *Neisseria* in saliva is associated with periodontal health ^65^ and the abundance of *Rothia* decreased in periodontitis. ^17^ Both bacteria are essential for reducing oral nitrate. ^53, 54^ Another study found that while the overall diversity of the oral microbiome declined in mice with diabetes mellitus, *Proteobacteria* along with *Firmicutes* numbers increased. ^66^ The information support our findings that elevated *Proteobacteria* abundance, with *Neisseria* being the most prevalent, seemed to be triggered by inflammation of the periodontal tissue in people with type 2 diabetes. However, we recommend more studies to validate these findings.

Since *Actinomycetota*’s *Rothia* are known NO ^-^_3_ reducers, ^67^ the lower salivary nitric oxide content indicated by the Griess reaction could be the reason why our T2DM patients with periodontal disease (G2 and G3 groups) had a smaller number of these bacteria than those without periodontal disease (G1 group). Nonetheless, the nitrate concentration of the G2 and G3 groups were comparable. This suggests that the advancement of periodontal disease (gingivitis and periodontitis) may impair the activity of nitrate reducer in either periodontal inflammation conditions. ^16^ Interestingly, groups G2 and G3 displayed a decrease in salivary nitrate concentration despite a higher number of *Neisseria.* Hence, this study indicate that there is a complex interaction between different bacterial species and their metabolic activity in the oral microbiome of our T2DM subjects, which is not covered in our study. Indeed, additional study is required to ascertain whether the increased *Neisseria* proportion in the microbiome of T2DM subjects with gingivitis or periodontitis causes or results from periodontal inflammation.

In addition to the microbial changes, our results might possibly be connected to the oxidative stress and systemic inflammation pathways that define diabetes and periodontitis. Reactive oxygen species (ROS) are produced by the host’s immunological response to oral infections in periodontitis, a chronic inflammatory disease that causes tissue damage and oxidative stress. ^68^ By producing systemic oxidative stress, which in turn interferes with insulin signaling, the inflammatory burden of the oral cavity may make glycemic control worse. ^69^ This cycle of inflammation and oxidative stress may be exacerbated by our G3 group’s greater abundance of periodontopathogens and lower amounts of nitrate-reducing bacteria like *Rothia*. The low salivary nitrite levels and the different microbial relationships we found between the gingivitis and periodontitis groups imply that the oral microbial dysbiosis in these patients may be a contributing factor to the increased oxidative stress and inflammation observed in both conditions.

It should be mentioned that the observed variation in bacterial counts between the gingivitis and periodontitis groups in our T2DM participants is not necessarily indicative of a biomarker’s diagnostic value. ^70^

Literature shows, that periodontopathic bacteria ( *P. gingivalis*, *T. denticola*, *T. forsythia*, and *Fusobacterium* spp.) are responsible for the development and progress of periodontal disorders. ^71^ The study’s findings clearly showed that people with type 2 diabetes accompanied with gingivitis or periodontitis have reduced nitrate reduction efficiency, with the exception of *Neisseria*, which affects the ability of microorganisms to reduce nitrate in saliva. ^16^ Therefore, the shifting of oral microbial equilibrium, particularly the balance of nitrate-reducing bacteria activities, may be the cause of periodontal inflammation in our diabetic subjects. Furthermore, a previous study demonstrated that the activity of bacteria that reduce nitrate may help minimize the risk of systemic diseases like hypertension and insulin resistance. Our descriptive finding suggest an association where these bacteria are more prevalent in gingivitis prior to periodontitis. Nevertheless, more study is required to validate this finding.

### Limitation

First, the study’s cross-sectional design makes it challenging to identify causal links. Furthermore, we accept that the unequal number of participants may be regarded as a limitation and that the sample size for each group was not established through a formal calculation. Confirming our findings would benefit from bigger, more balanced cohort studies in the future. Second, the pooling of all genomic DNA samples into a single library per group renders the metagenomic results strictly descriptive and exploratory. This method precludes the use of inferential statistics and the determination of statistically significant differences between the groups. ^8^ Nonetheless, the findings confirm and reinforce the results of the 16S rRNA gene sequencing analysis.

Additionally, we acknowledge that the absence of critical systemic data, including BMI, HbA1c levels, and duration of T2DM, is a significant limitation. The different levels of glycemic control among participants may have affected the microbial profiles, yet our study was limited to a representative sample of T2DM patients. In order to better examine this association, future research could benefit from grouping participants according to their HbA1c levels.

Furthermore, the radiographic evaluation for assessing alveolar bone loss, was not included in our investigation. Accurate disease staging was made possible by our use of clinical indicators (CAL and probing depth), but we acknowledge that further research integrating our microbiological results with radiographic data may offer a more complete view of the progression of the disease.

Lastly, we did not include smoking habit, which may be a confounder in the study results. However, data are emerging that the oral microbiota, which is associated with periodontal disease, may be strongly correlated with the incidence of type 2 diabetes, even when confounders are excluded.

## Conclusion

The study’s findings showed that there are notable variations in the microbial communities in saliva between periodontal disease and oral health, with the development of periodontal inflammation in diabetics playing a substantial role in these differences. Overall, the results show that people with type 2 diabetes mellitus who also have periodontitis or gingivitis may have unique relationships between nitrate-reducing and periodontopathic bacteria in their salivary microbiome. In order to prevent periodontitis, these characteristics may be promising microbiological features that warrant validation for the early identification and treatment of gingivitis.

## Author contributions

BB: Data curation, Funding acquisition, Writing-review & editing. DT: Validation, Visualization, Review & editing. CT: Resources, Supervision, Review &editing. NH:, Resources, Validation. CT: Project administration, Validation.YS: Data curation, Validation. SS: Validation, Visualization, Review & editing. AW: Data curation, Validation. FR: Resources and Supervision FT: Data curation, Validation, Visualization. DA: Resources and Supervision. EB: Conceptualization, Writing-Original draft.

## Ethics and consent

In accordance with the criteria of the institutional ethics committee, All participants provided written informed consent before they participated part in the study, and the Dr. Cipto Mangunkusumo Hospital’s Ethics Committee approved the study’s protocols (Ethics Reference Number: KET-1203/UN2.F1/ETIK/PPM.06.02/2023).

## Data Availability

Figshare: Raw data salivary microbiome using 16s barcoding kit 24 V14, ONT (Oxford Nanopore Technology), https://doi.org/10.6084/m9.figshare.28365782.v4 ^72^ This project contains the following underlying data:
• FASTQ files in folder of barcode 18, 19, 20 • Subject data in.xlsx format •
Figures in PNG and JPG format Data are available under the terms of the Creative Commons Zero “No rights reserved” data waiver (CC0 1.0 Public domain dedication).
