# Salivary microbiome and periodontopathogen/denitrifying bacteria associated with gingivitis and periodontitis in people with type 2-diabetes

**DOI:** 10.12688/f1000research.161731.4

**Published:** 2026-01-13

**Authors:** Endang Bachtiar, Boy M. Bachtiar, Dicky L Tahapary, Turmidzi Fath, Citra Fragrantia Theodora, Natalina Haerani, Selvi Nafisa Shahab, Yuniarti Soeroso, Ardy Wildan, Fergie Marie Joe Grizella Runtu, Fatimah Maria Tadjoedin, Dewi Ayuningtyas

**Affiliations:** 1 Oral Biology Fac. of Dentistry, University of Indonesia, Depok, West Java, 16424, Indonesia; 2 Clinical Research Unit RSCM, . Metabolic-Endocrine-Diabetes Division, Dept. Internal Medicine, Universitas Indonesia, Depok, West Java, Indonesia; 3 Faculty of Dentistry, Department of Oral Biology and Oral Science Research Center, Universitas Indonesia, Depok, West Java, 10430, Indonesia; 4 Faculty of Dentistry,Department of Periodontology, Universitas Indonesia, Jakarta, Jakarta, 10430, Indonesia; 5 Faculty of Medicine, Department Microbiology, Clinical Research Unit RSCM, Universitas Indonesia, Ciptomangunkusumo Hospital., West Java, Indonesia; 6 Metabolic-Endocrine-Diabetes Division, Dept. Internal Medicine, Universitas Indonesia, Depok, West Java, Indonesia

**Keywords:** Diabetes; gingivitis; periodontitis; salivary microbiome; nanopore sequencing

## Abstract

**Background:**

Despite diabetes mellitus and periodontal diseases are mutually exclusive, little is known about particular types of bacteria that may have exacerbated the development of diabetics’ periodontal inflammation. The purpose of this study was to descriptively characterize and explore the differences in the salivary microbiomes of individuals with type 2 diabetes (20-40 years old) who had gingivitis or periodontitis to those who did not. Additionally, we evaluated the descriptive relationship between the relative abundance of periodontopathogens and nitrate-reducing bacteria in their salivary microbiome.

**Methods:**

Saliva was collected from all participants. Genomic DNA was isolated and pooled in equimolar quantities from all individuals within each group to create three pooled libraries: type 2 diabetes (T2DM) patients without periodontal disease (G1), T2DM patients with gingivitis (G2), and T2DM patients with periodontitis (G3). Sequencing was performed using Oxford Nanopore MinION Technology. The relative abundance and bacterial diversity were measured using bioinformatic methods, and all analyses of sequencing data were strictly descriptive and exploratory. Salivary nitrite/nitrate concentrations were measured on individual, un-pooled samples.

**Results:**

The salivary microbiota among people with type 2 diabetes and periodontal disease (G2; G3) was observed to have greater bacterial diversity and abundance than that of patients without periodontal disease (G1), according to descriptive alpha-diversity analysis. The G3 group exhibited the largest relative abundance of *Porphyromonas gingivalis*, a key periodontopathogen. Descriptive analysis also suggested that periodontopathic bacteria and nitrate-reducing bacteria have different community structures across the groups. Furthermore, comparison of individual salivary samples showed that nitrite/nitrate concentration was significantly lower in the G3 group compared to the G1 group (p< 0.05). Results reveal that the inverse interaction between increased *P. gingivalis* and decreased nitrate-reducing bacteria serves as a descriptive hallmark for periodontal disease progression in the T2DM population.

**Conclusion:**

Results of this exploratory study suggest that the relationship between periodontopathic and denitrifying bacteria in the salivary microbiome varies among those with type 2 diabetes mellitus who also have gingivitis or periodontitis. These distinct microbial features observed may be microbiological characteristics associated with the progression of periodontal disease in this population, warranting further validation as potential indicators for early management.

## Introduction

Maintaining periodontal health requires a balanced microbial community where specific functional groups play distinct and antagonistic roles. Periodontopathogens, particularly the “red complex” led by *Porphyromonas gingivalis*, are well-characterized for their ability to bypass host immune defenses and initiate tissue destruction through the release of various virulence factors. ^1^ ^,^ ^2^ Conversely, nitrate-reducing bacteria (NRB), such as *Rothia, Neisseria, Actinomyces, Veillonella, Kingella, Propionibacterium, Prevotella, Granulicatella,* and *Haemophilus*, serve as essential components of the oral ecosystem due to their crucial role in the nitrate (NO _3_) – nitrite (NO _2_) – nitric oxide (NO) pathway of the human nitrogen cycle. ^3^ ^,^ ^4^ NO acts as a potent antimicrobial and signaling molecule that helps maintain vascular tone and suppresses the overgrowth of orally acquired pathogens. ^5^ ^,^ ^6^

The homeostatic balance between periodontopathogens and NRB may be significantly disrupted by systemic metabolic alterations, particularly those linked to Type 2 Diabetes Mellitus (T2DM). Recent studies indicate that individuals with T2DM are more likely to develop periodontitis and suffer from more severe forms of the disease. ^7^ ^–^ ^9^ Although numerous studies have explored the bidirectional relationship between diabetes mellitus and periodontal disorders, there are still gaps in the literature regarding how T2DM influences the ecological shift between these two specific bacterial groups. ^10^ ^–^ ^12^

In diabetic patients, hyperglycemia and increased oxidative stress are thought to alter the oral environment, potentially favoring the proliferation of anaerobic pathogens. ^13^ ^,^ ^14^ This dysbiosis is not only characterized by the presence of pathogens but also by the depletion of beneficial bacteria. ^15^ Specifically, it is suggested that the unique metabolic environment of T2DM facilitate disease progression via a functional shift - a disruption in the beneficial NO-production function of the NRB community – which is closely associated with an increased abundance of periodontopathic species. ^3^ ^,^ ^16^

Beyond identifying individual microbial taxa, it is crucial to understand the ecological interplay between these groups in the context of disease progression from gingivitis to periodontitis. In this study, saliva was chosen as the sample type to accurately capture the oral microbiome’s composition, as it effectively reflects the microbial shifts that occur during periodontal destruction. ^17^ ^,^ ^18^ Furthermore, the Oxford Nanopore Technology (ONT) long-read sequencing approach was employed to provide species-level identification based on whole 16S rRNA sequences. ^19^ ^,^ ^20^ By utilizing this high-resolution approach, this study aims to clarify how the interaction between periodontopathogens and nitrate-reducing bacteria relates to the severity of periodontal destruction in patients with T2DM.

## Methods

### Participants and patient characteristic

Participants in this cross-sectional study were Indonesian adult patients recruited from the Dr. Cipto Mangunkusumo Hospital in Jakarta between November 2023 and January 2024. Participants were selected to have at least 20 teeth without any obvious evidence of root or oral mucosal caries, be within the ages of 20 and 40, and have a diagnosis of non-insulin-dependent Type 2 Diabetes Mellitus diagnosed by the internist of the Division of Endocrinology, based on the conditions of blood glucose level 2 hours after oral glucose load ≥ 200 mg/dL, HbA1c ≥ 6.5%, or plasma glucose was ≥ 200 mg/dl with classic hyperglycemic crisis (not shown). In addition, participants had not smoked within three months, were not taking antibiotics or nonsteroidal anti-inflammatory medications, and had not had periodontal surgery or therapy within the preceding six months. Among the exclusion criteria were being pregnant or nursing, using specific drugs (such hormones) within six months of sample collection, or having a metabolic disorder other than diabetes. Two competent and experienced periodontists assessed each participant’s periodontal health. According to current classification guidelines, the diagnosis was only made based on clinical features like Clinical Attachment (CAL > 2 mm) and Probing Depth (PD > 4 mm). We recognize that our review did not take radiographic evidence of alveolar bone loss into account. The examiner was calibrated before starting the investigation to guarantee measurement reliability and consistency. The inter-examiner reliability was evaluated using the Kappa test, with a value of 0.86.

The diagnosis of gingivitis was made by bleeding on probing (BOP) score, ^21^ while chronic periodontitis was diagnosed based on the standard classification of the America academic of periodontology, ^22^ without radiological evaluation to support the use of the North Caroline periodontal probe (UNC-15). Participants diagnosed with chronic periodontitis were identified as having at least 30% of sites with alveolar bone resorption, as well as more than 4 sites with probing depth (PD) ≥ 4 mm and clinical attachment loss (CAL) ≥ 2 mm.

In accordance with the criteria of the institutional ethics committee, all participants provided written informed consent before they participated part in the study, and the Dr. Cipto Mangunkusumo Hospital’s Ethics Committee approved the study’s protocols (Ethics Reference Number: KET-1203/UN2.F1/ETIK/PPM.00.02/2023).

Data availability note: Clinical data on Body Mass Index (BMI), HBA1c levels, and duration of T2DM were not available for inclusion in this manuscript due to institutional privacy and ethical agreements with the collaborating Faculty of Medicine Universitas Indonesia.

### Saliva sample collection, DNA extraction, and sequencing

Following a protocol described elsewhere, ^23^ after rinsing their mouths, using 0.9% normal saline for about 30s, approximately 3-5 mL of unstimulated whole saliva was collected from participants, one hour after they ceased eating, drinking, or brushing their teeth, and prior to any clinical periodontal assessment to prevent potential interference from bleeding or mechanical irritation. For DNA analysis, 1.5 mL of the sample was immediately treated with DNA stabilization buffer, and genomic DNA was isolated. The remaining saliva was immediately aliquoted for chemical analysis and stored at -80°C until analysis. These aliquoted saliva samples were maintained individually and were not pooled.

DNA was extracted using the Monarch ^®^^,^™ Genomic DNA purification kit, NEB #T3010S/L (New England Biolabs, Bruningstrasse Frankfurt am Main, Germany) and quantified using a Qubit 2.0 Fluorometer (Invitrogen, Carlsbag, CA, USA). The inclusion of negative controls, or no-template controls, in the DNA extraction and PCR amplification processes allowed us to verify the validity of our findings by looking for contamination. To further validate the effectiveness of the PCR reaction, a positive control that came with the 16S Barcoding Kit was utilized.

DNA pooling and sequencing: Furthermore, the genomic DNA of each study group’s samples was ligated in equimolar quantities to create a pool of three group libraries for nanopore sequencing. Each barcoding kit contained 50 ng of starting DNA. Nanopore amplicon library was prepared using the 16S Barcoding Kit 24 V14 (SQK-RAB204, Oxford Nanopore Technologies, UK) following the manufacturer’s instruction. Primers 27F and 1492R are included in the kit to amplify the whole 16S rRNA gene.

Sequencing was conducted using the MinION (Oxford Nanopore Technologies, UK) with a MinION flow cell (R10.4.1) for 8 hours. Subsequently, the basecalling were generated using MinKNOW (Oxford Nanopore Technologies, UK). For microbiota profiling analysis, we followed the EPI2ME for wf-metagenomic workflow for real time analysis. The analysis results were further generated in the form of a report in the EPI2ME for wf-metagenomic.

Filtering was implemented before the generation of the relative abundance table. Raw sequencing data were base-called utilising Dorado (v7.6.8), and reads were trimmed and filtered according to a minimal quality score (Q-score ≥ 8). The resulting pass reads were further processed with the Nextflow wf-metagenomics pipeline, and only reads within the length range of 200–1500 bp were included for taxonomic classification using Kraken2 (ncbi_16s_18s_28s_ITS database). As our focus was on reporting the relative abundances derived from high-quality filtered reads. Only the several abundant genera or species (periodontopathogen/denitrifying bacteria associated with gingivitis and periodontitis) were displayed, while the others were grouped as ‘Others’.

### Bioinformatic preprocessing and descriptive analysis (pooled DNA)

For microbiota profiling analysis (OTU, alpha diversity, and rarefaction curve), the EPI2ME for wf-metagenomic was used. To ensure data quality, only high-quality reads (“pass”, >5 cumulative reads) were included in the analysis. ^24^ Raw sequencing data were base-called utilising Dorado (v7.6.8), and reads were trimmed and filtered according to a minimal quality score (Q-score ≥8). The resulting pass read were further process with the netflow wf-metagenomics pipeline, and taxonomic classification using Kraken2 (ncbi_16s_18s_28s_ITS database). The operational taxonomic units (OTUs) in each group and the rarefaction curve were developed using EPI2ME, and analysed using RStudio 4.3.2.

The analysis of the pooled sequencing data, including alpha diversity (Simpson and Shannon indices) and taxonomic comparisons, was strictly descriptive and exploratory. No inferential statistic (such as Kruskal-Wallis test, t-test, or one way ANOVA) were applied to the sequencing data, as the pooling of samples precludes the estimation of inter-individual variance. Only the several abundant genera or targeted species were displayed, while others were grouped as “Others”.

### Salivary nitrite/nitrate measurement and inferential analysis

Aliquoted individual saliva samples were thawed, and the total salivary nitrite/nitrate concentrations were quantified for each participant using Griess Reagent System (Promega #TB229, Madison, WI 53711-5399 USA), ^25^ allowing the mixture remain at room temperature for 10 minutes in the dark, and then using a spectrophotometer (AccuReader. M965/M965+, Nangang, Taipei, Taiwan), to quantify the mixture at 450 nm.

For salivary nitrite/nitrate levels, which were measured on individual participants, descriptive statistics are presented as Mean ± Standard Deviation (SD). Differences in mean values were determined by one-way ANOVA test or Kruskal-Wallis test. GraphPad Prism software version 10 was used to perform these analyses. A significance level of p< 0.05 was employed for all inferential tests performed on the individual-level data.

## Results

### Patient characteristics and clinical parameters

The study included n = 171 participants (G1: n = 28; G2: n = 54; G3: n = 89). Baseline demographic and clinical characteristics are presented in Table 1. There were no statistically significant differences observed between the groups for Age and Sex (p > 0.05). Clinical periodontal parameters, including Mean Probing Depth (PD), Clinical Attachment Loss (CAL), and Bleeding on Probing (BOP), were statistically different across the three groups, reflecting the disease status used for grouping ( Table 1). Data on BMI, HbA1c, and T2DM duration were not available for inclusion in this study.

**Table 1.** Baseline demographic and clinical characteristics of study. Characteristics are shown as Mean ± Standard Deviation (SD) for continuous variables and as absolute number (n) and percentage (%) for categorical variables (Sex). Differences between the three study groups were assessed using one-way ANOVA for parametric data (Age) and the Kruskal-Wallis test for non-parametric variables (PD, CAL, % Sites with BOP). Group 1 (G1) = No periodontal disease, Group 2 (G2) = Gingivitis, and Group 3 (G3) = periodontal disease.

Characteristic	No Periodontal disease (G1)	Gingivitis (G2)	Periodontitis (G3)	p-value
**Total participants, n (%)**	n = 29 (17%)	n = 54 (31%)	n = 89 (52%)	-
**Age (years), Mean ± SD**	40.94 ± 7.64	43.3 ± 10.3	50.07 ± 8.84	p > 0.05
Male, n (%)	16 (55.2%)	24 (44.4%)	40 (44.9%)	p < 0.05
Female, n (%)	13 (44.8%)	30 (55.6%)	49 (55.1%)	p < 0.05
**Probing depth (PD, mm)** **Mean ± SD**	2.4 ± 0.1	2.6 **±** 0.3	4.8 **±** 0.2	p < 0.05
**Clinical attachment loss** **(CAL, mm), Mean ± SD**	0.14 ± 0.1	0.2 ± 0.1	2.2 ± 0.2	p < 0.05
**% Sites with BOP** **Mean ± SD**	-	9.7 ± 1.4	10 ± 1.3	p < 0.05

### Microbial diversity and Phylogenetic Profile

**Figure 1.**
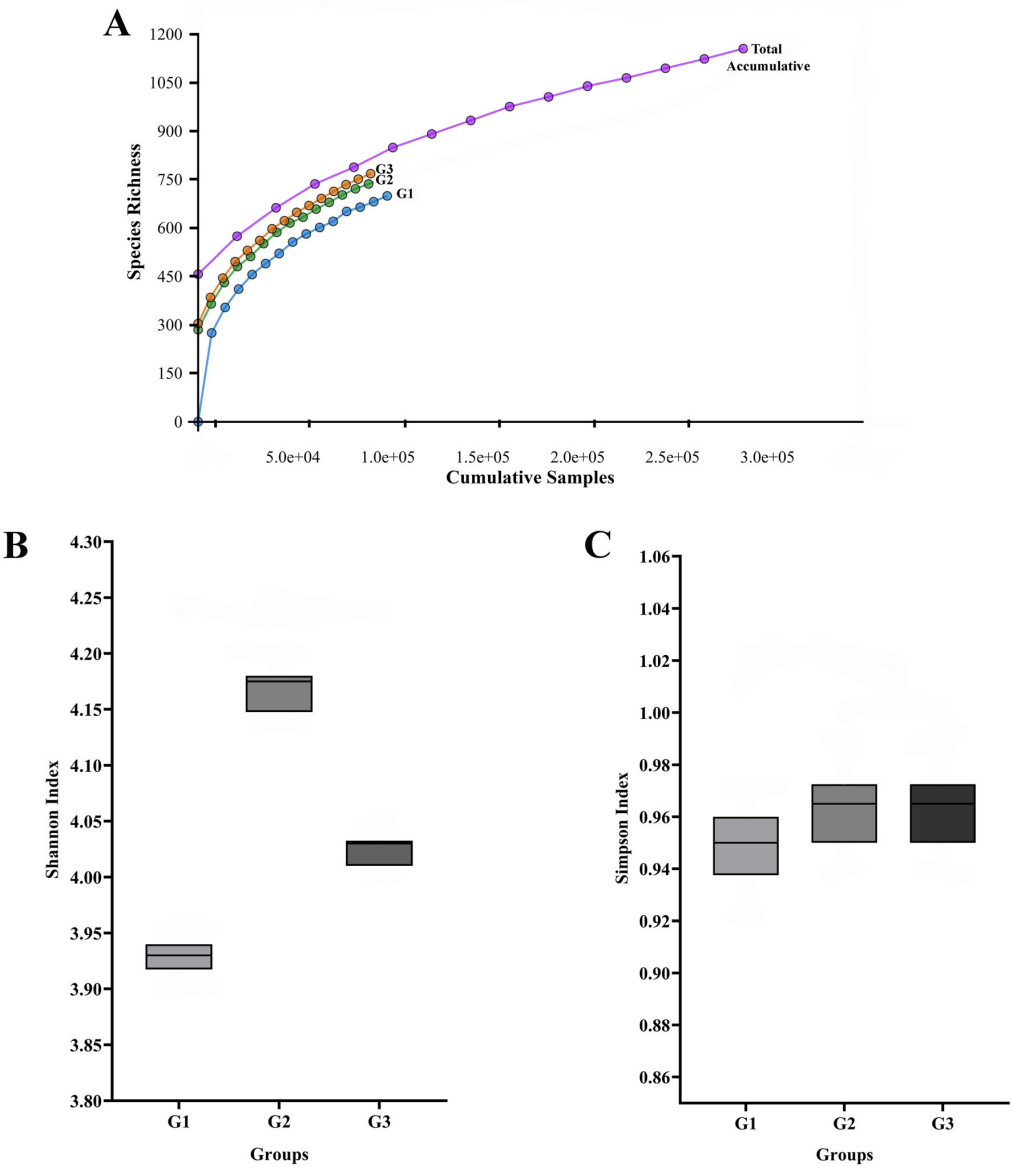
The salivary microbiome's rarefaction curve and alpha diversity among different T2DM patient groups. The total number of sequences is displayed on the horizontal axis, while the number of operational taxonomic units (OTUs) at a 97% intersequence similarity level is shown on the vertical axis of the rarefaction curve (A). The diversity indices of Shannon (B) and Simpson (C) show how alpha diversity varies among the three groups in terms of evenness and richness. Species diversity and evenness seem to be larger in gingivitis group (G2) and periodontitis group (G3) compared to oral health group (G1).

The rarefaction curves ( Figure 1A) for all three pooled libraries (G1, G2, and G3) reached an asymptote, indicating that the sequencing depth was sufficient to capture the majority of the variation present in the pooled samples.

Descriptive alpha diversity analysis using the Shannon and Simpson indices showed a tendency toward higher species richness and evenness when comparing the pooled G2 and G3 libraries to the pooled G1 library ( Figure 1B, 1C). The differences were especially apparent when comparing the G1 group (No periodontal disease) to the G3 group (periodontitis).

**Figure 2.**
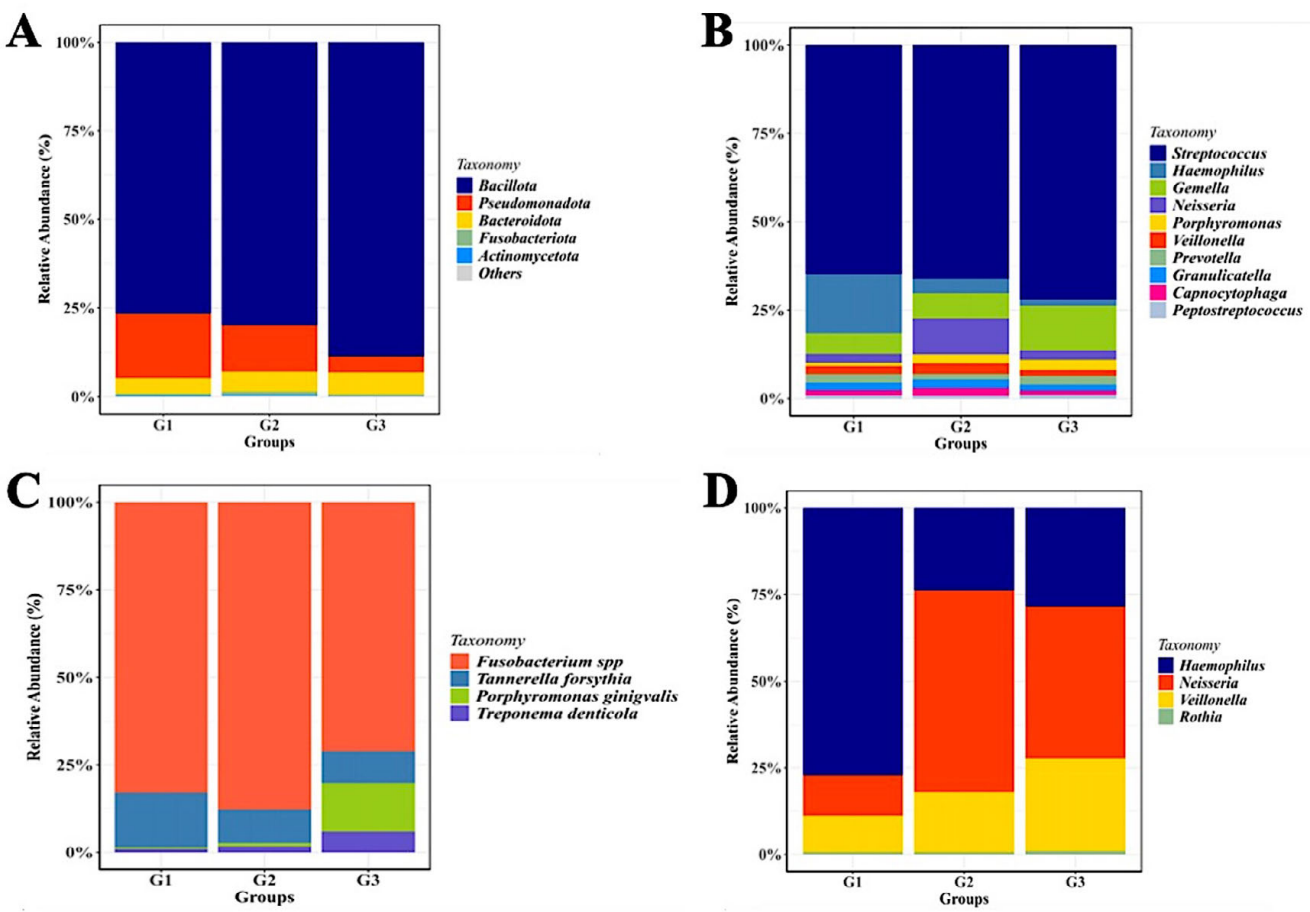
Saliva microbiota taxonomic composition in T2DM patients with and without periodontal diseases. Relative abundance of mayor of salivary bacterial taxa, which relative abundance >5%, are presented at the phylum (A) and genus (B) level. “Others” refers to the remaining phyla. A comparison of the relative abundance of periodontopathic bacterial species (C) and genus of NO _3_^-^-reducing bacteria (D) between three groups of patient with T2DM. G1 through G3 represent the participant group.

**Figure 3.**
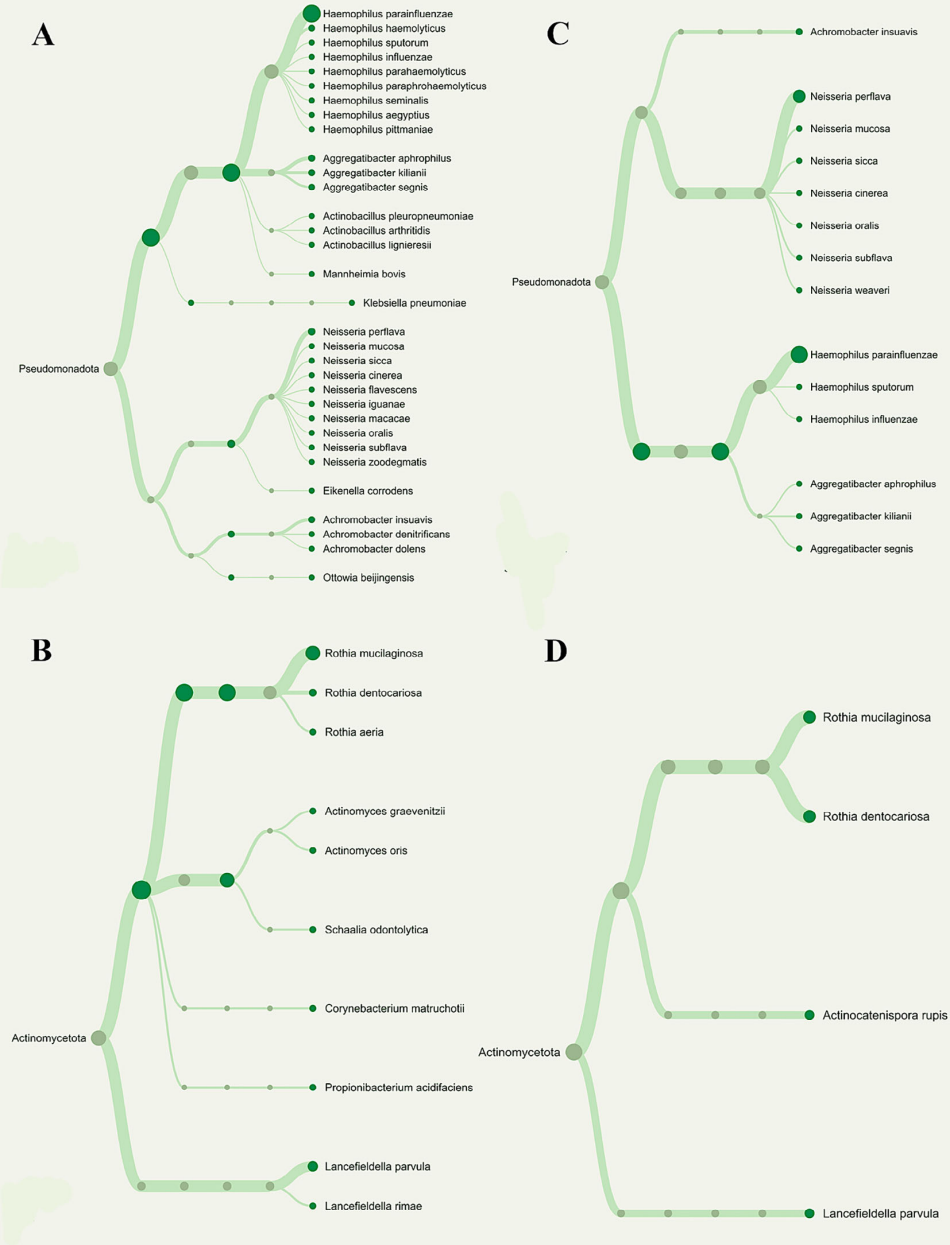
Phylogenetic variations among nitrate-reducing bacteria between T2DM patients with periodontitis (G3) and gingivitis (G2). Overall phylogenetic diversity of nitrate-reducing bacteria was considerably lower in T2DM individuals with periodontitis than in those with gingivitis. Patients with periodontitis (A and B) or gingivitis (C and D), but not both, have specific *Neisseria* species (Blue box). The distribution of *Rothia* species (Red box) also differed among the groupings.

The number of Operational Taxonomic Units (OTUs) found in each pooled group is displayed in Figure 2, and the phylogenetic analysis ( Figure 3) demonstrates the general taxonomic structure and relationships of the observed microbial communities. These diversity measures were not subjected to inferential statistics.

The sequencing analysis revealed a dynamic shift in the salivary microbial composition across the three groups. As shown in Figure 2C, although *P. gingivalis* is a primary pathogen, our results indicated that *Fusobacterium* spp. exhibited a higher relative abundance in the gingivitis (G2) and periodontitis (G3) groups compared to the healthy group (G1). Regarding the nitrate-reducing bacteria (NRB) community, the distribution of *Neisseria* showed a specific pattern ( Figure 2D). Interestingly, the proportion of *Neisseria* was observed to be lower in the healthy T2DM group (G1) compared to the gingivitis (G2) and periodontitis (G3) groups.

Although the phylum-level microbiome is dominated by *Firmicutes*, the relative abundance of certain genera varies significantly amongst the groups. *Streptococcus* was the most prevalent taxa in each group. *Haemophilus* was most prevalent in group G1 (14%), followed by groups G2 and G3 (3% and 1.5%, respectively). *Neisseria* (22%) and *Gemella* (24%) were two other prominent genera that were shown to be abundant in the G2 and G3 groups, respectively. Although the three groups’ relative abundances of *Porphyromonas* seem to vary, group G3 still has higher numbers of these genera than the other two groups.

### Relative abundance and key taxa

**Figure 4.**
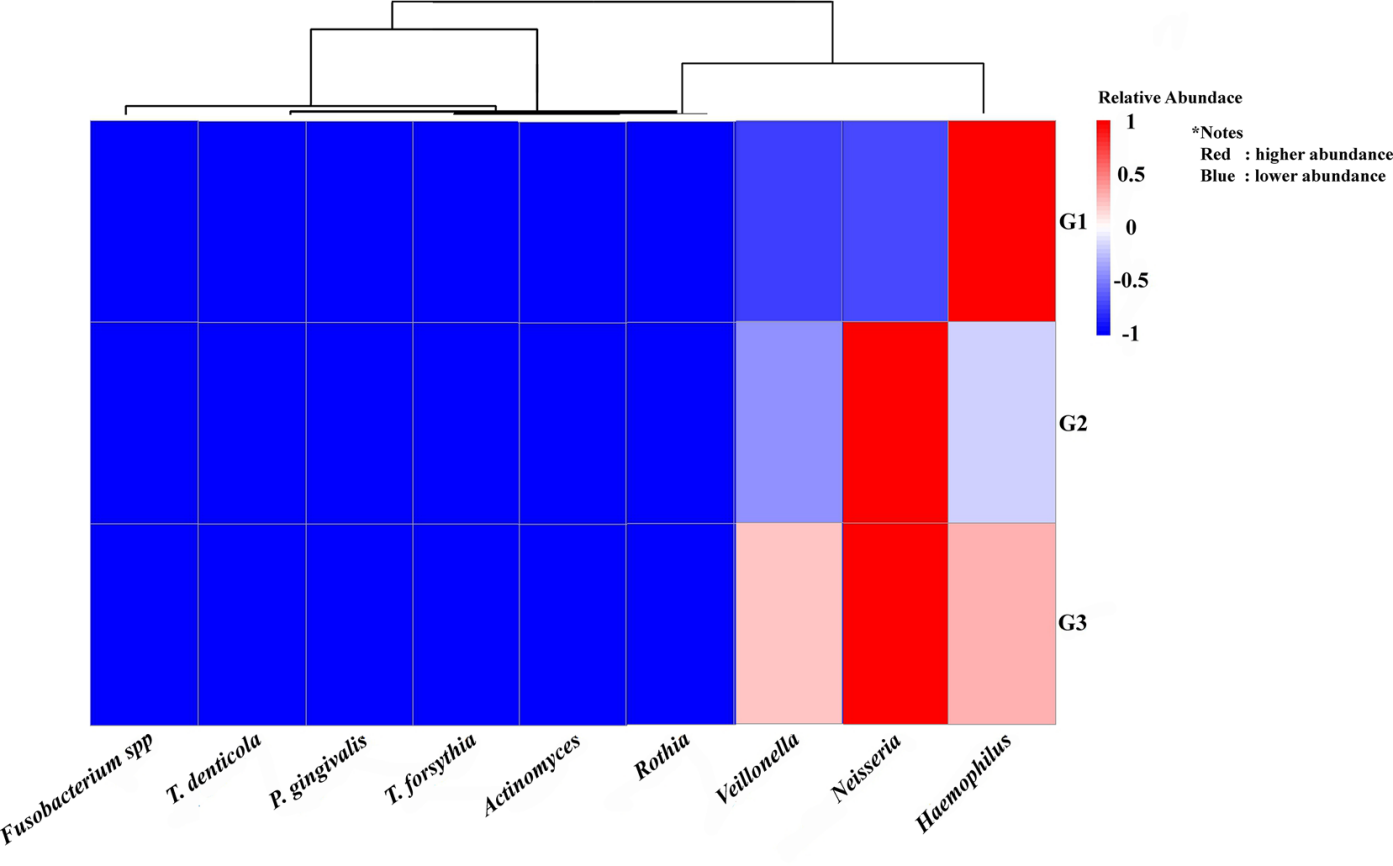
The heatmap displays the quantity of periodontopathic and nitrate-reducing bacteria in the groups under study. The study groups and the targeted bacteria were represented by rows and columns, respectively. The relative proportion of the bacterial assignment within each group is represented by the colors in the heatmap. A shift in color toward dark red denotes a higher abundance.

As seen by the Heat Map ( Figure 4), descriptive analysis of the relative abundance profiles showed distinct variations in the taxonomic structure across the three pooled groups. The highest relative abundance of key periodontopathogens, including Porphyromonas gingivalis and Treponema denticola, was observed in the pooled periodontitis library (G3). However, more beneficial nitrate-reducing bacteria (NRB) taxa, like Neisseria and Rothia, seemed to be prevalent in the combined G1 and G2 libraries. A descriptive comparison revealed a distinct and increasing trend in the periodontopathogen-to-NRB ratio from the G1 to the G3 groups.

This figure’s data are all descriptive relative abundances.

### Salivary nitrite/nitrate concentration (individual data)

**Figure 5.**
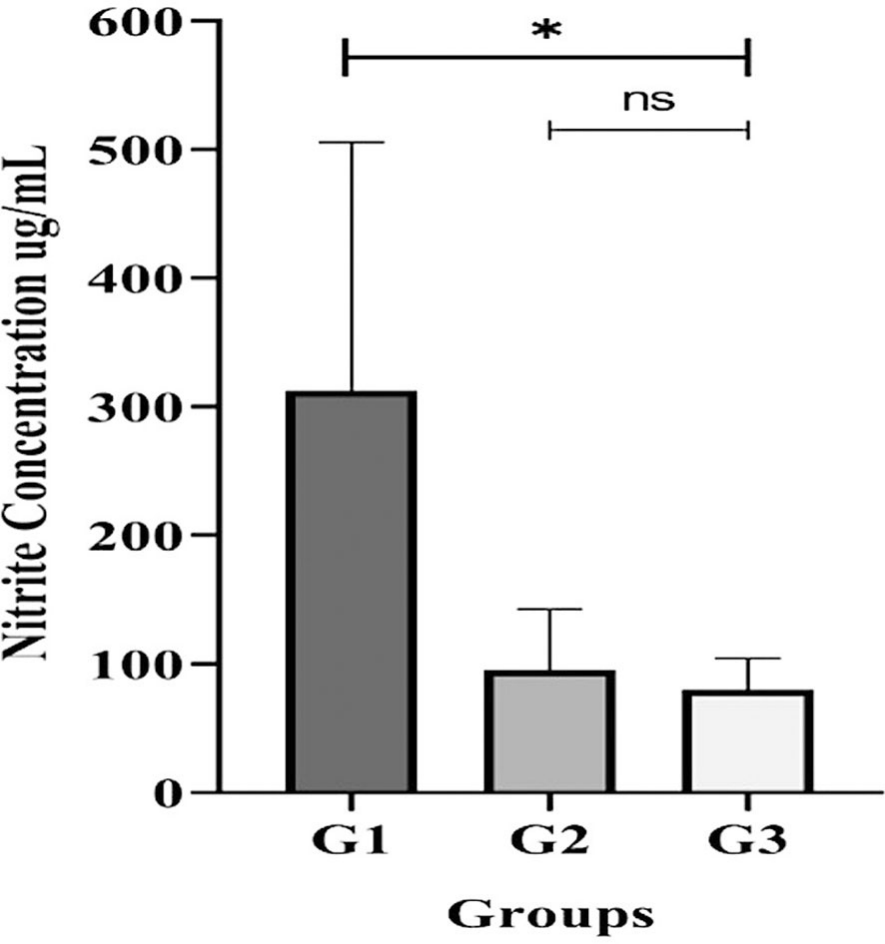
Salivary nitrite concentration in unstimulated saliva from T2DM subject groups. All participants with gingivitis (G2), periodontitis (G3), and no periodontal disease (G1) had their salivary nitrite levels measured using the Griess reaction method. Bars represent mean + SD. An asterisk (*) indicates a statistically significant difference (p-value < 0.05) and ns = not significant.

Analysis of individual, un-pooled saliva samples revealed significant differences in the total salivary nitrite/nitrate concentrations between the groups ( Figure 5). The G3 (periodontitis) group had a significantly lower concentration (p < 0.05) based on the Kruskal-Wallis test.

## Discussion

The present study demonstrates that the salivary microbiome of T2DM patients undergoes a significant ecological shift as periodontal disease progresses. Our findings suggest a profound inverse relationship between the abundance of periodontopathogens and nitrate-reducing bacteria (NRB). This shift is not merely a change in individual taxa but reflects a disruption of the homeostatic balance required for oral health. In the unique metabolic environment of T2DM, characterized by hyperglycemia and oxidative stress, this dysbiotic transition appears more pronounced, as the systemic condition potentially facilitates a more hospitable environment for anaerobic pathogens while suppressing beneficial commensals. ^13^ ^,^ ^14^ ^,^ ^26^

First, we found that the 16S rRNA-based MinION technology’s sequencing depth allowed for the identification of 97% of the bacterial population, allowing the ability to identify bacterial cells in pooled saliva samples. Subsequently, these studies’ findings showed that T2DM patients with periodontal diseases were observed to have a greater alpha diversity in their saliva than those without the disease. The result suggests that the salivary microbiota’s composition significantly shifted from symbiosis to dysbiosis as our subjects’ periodontal health deteriorated. The findings may also imply that the diversity of individual microbial patterns among our diabetes group members may have led to the difference in alpha diversity seen in this study. Additionally, using the Shannon and Simpson indices, we descriptively found that T2DM participants with gingivitis (G2 group) and periodontitis (G3 group) had higher species variety than those without periodontal diseases (G1 group). However, people with T2DM and gingivitis may have more low-abundance bacterial species in their saliva, which could explain the higher Shannon index. Although they have little effect on the Simpson’s index, these rare species add to the total diversity measured by the Shannon index. This implies that, despite the fact that gingivitis and periodontitis displayed different clinical symptoms, species abundance rather than species diversity is the primary factor influencing the observed differences in salivary microbiome between the two groups. Further research is necessary to fully understand the implications of these findings and their potential therapeutic relevance. However, it should be remembered that these diversity shifts may not always be associated with changes in the relative abundances of the microbiome; they may instead be explained by certain ecological conditions that influence the patterns of microbial succession. ^27^

A key observation in our study is the proportion of *P. gingivalis* and *Fusobacterium* spp. relative to the NRB community. While *P. gingivalis* is recognized as a keystone pathogen in periodontitis, our data ( Figure 2C) showed a high prevalence of *Fusobacterium* spp. in certain groups. This interaction is critical, as *Fusobacterium* acts as a bridge organism that facilitates the colonization of other pathogens, including *P. gingivalis*, thereby exacerbating the inflammatory response in the periodontal tissues of diabetic patients. ^15^ ^,^ ^16^ The observed dominance of these pathogens correlates with the clinical severity of attachment loss and probing depth seen in groups G2 and G3. Moreover, the specific microbial profile in the saliva of diabetic patients indicates a complex ecological transition. The results suggest that the progression of periodontal destruction in T2DM individuals is characterized by a significant fluctuation in the ratio between periodontopathogenic species and the nitrate-reducing community, particularly involving *P. gingivalis, Fusobacterium* spp., and *Neisseria*.

Furthermore, we addressed the specific role of the NRB community, such as *Neisseria* and *Rothia*. Theoretically, a healthy oral environment is supported by high levels of NRB, which maintain the nitrate-nitrite-nitric oxide (NO) pathway to suppress pathogens. ^3^ ^,^ ^4^ Interestingly, our results ( Figure 2D) showed a specific pattern for *Neisseria* across the groups. The reduction of nitrate-reducing bacteria in T2DM patients may impair these protective nitric oxide-mediated pathways, thereby diminishing the host’s ability to inhibit the overgrowth of anaerobic pathogens. ^25^ This ecological interaction-where the decline of NRB occurs alongside the expansion of the “red complex”—serves as a comprehensive descriptive indicator of periodontal destruction in the T2DM population.

The bidirectional link between T2DM and periodontitis further complicates this microbial interplay. Systemic inflammation from diabetes likely influences the salivary environment, providing specific substrates that favor a pathogenic shift. ^7^ ^,^ ^10^ By understanding these interactions rather than focusing on single pathogens, we gain a better perspective on how periodontal disease is sustained in diabetic individuals. These findings emphasize that maintaining the nitrate-reducing capacity of the oral microbiome could be as important as reducing pathogen load in managing periodontal health for those with T2DM. ^28^ ^,^ ^29^

Literature shows, that periodontopathic bacteria ( *P. gingivalis*, *T. denticola*, *T. forsythia*, and *Fusobacterium* spp.) are responsible for the development and progress of periodontal disorders. ^30^ The study’s findings clearly showed that people with type 2 diabetes accompanied with gingivitis or periodontitis have reduced nitrate reduction efficiency, with the exception of *Neisseria*, which affects the ability of microorganisms to reduce nitrate in saliva. ^28^ Therefore, the shifting of oral microbial equilibrium, particularly the balance of nitrate-reducing bacteria activities, may be the cause of periodontal inflammation in our diabetic subjects. Furthermore, a previous study demonstrated that the activity of bacteria that reduce nitrate may help minimize the risk of systemic diseases like hypertension and insulin resistance. Our descriptive finding suggest an association where these bacteria are more prevalent in gingivitis prior to periodontitis. Nevertheless, more study is required to validate this finding.

### Limitation

First, the study’s cross-sectional design makes it challenging to identify causal links. Furthermore, we accept that the unequal number of participants may be regarded as a limitation and that the sample size for each group was not established through a formal calculation. Confirming our findings would benefit from bigger, more balanced cohort studies in the future. Second, the pooling of all genomic DNA samples into a single library per group renders the metagenomic results strictly descriptive and exploratory. This method precludes the use of inferential statistics and the determination of statistically significant differences between the groups. ^31^ Nonetheless, the findings confirm and reinforce the results of the 16S rRNA gene sequencing analysis.

Additionally, we acknowledge that the absence of critical systemic data, including BMI, HbA1c levels, and duration of T2DM, is a significant limitation. The different levels of glycemic control among participants may have affected the microbial profiles, yet our study was limited to a representative sample of T2DM patients. In order to better examine this association, future research could benefit from grouping participants according to their HbA1c levels.

Furthermore, the radiographic evaluation for assessing alveolar bone loss, was not included in our investigation. Accurate disease staging was made possible by our use of clinical indicators (CAL and probing depth), but we acknowledge that further research integrating our microbiological results with radiographic data may offer a more complete view of the progression of the disease.

Lastly, we did not include smoking habit, which may be a confounder in the study results. However, data are emerging that the oral microbiota, which is associated with periodontal disease, may be strongly correlated with the incidence of type 2 diabetes, even when confounders are excluded.

## Conclusion

In conclusion, this study identifies a distinct ecological shift in the salivary microbiome associated with the severity of periodontal disease in patients with type 2 diabetes (T2DM). The progression from gingivitis to periodontitis is characterized by an intricate interaction between increased periodontopathogens, specifically *P. gingivalis* and *Fusobacterium* spp., and the shifting dynamics of nitrate-reducing bacteria such as *Neisseria* and *Rothia*. The imbalance in this microbial interplay – marked by the expansion of anaerobic pathogens and the disruption of beneficial nitrate-reducing communities-serves as a descriptive hallmark of periodontal tissue destruction in the T2DM population. These findings suggest that monitoring the interaction between these specific bacterial groups could provide valuable insights into the periodontal health status of individuals with diabetes.

## Author contributions

BB: Data curation, Funding acquisition, Writing-review & editing. DT: Validation, Visualization, Review & editing. CT: Resources, Supervision, Review &editing. NH:, Resources, Validation. CT: Project administration, Validation.YS: Data curation, Validation. SS: Validation, Visualization, Review & editing. AW: Data curation, Validation. FR: Resources and Supervision FT: Data curation, Validation, Visualization. DA: Resources and Supervision. EB: Conceptualization, Writing-Original draft.

## Ethics and consent

In accordance with the criteria of the institutional ethics committee, All participants provided written informed consent before they participated part in the study, and the Dr. Cipto Mangunkusumo Hospital’s Ethics Committee approved the study’s protocols (Ethics Reference Number: KET-1203/UN2.F1/ETIK/PPM.06.02/2023).

## Data Availability

Figshare: Raw data salivary microbiome using 16s barcoding kit 24 V14, ONT (Oxford Nanopore Technology), https://doi.org/10.6084/m9.figshare.28365782.v4 ^32^ This project contains the following underlying data:
• FASTQ files in folder of barcode 18, 19, 20 • Subject data in.xlsx format •
Figures in PNG and JPG format Data are available under the terms of the Creative Commons Zero “No rights reserved” data waiver (CC0 1.0 Public domain dedication).
