# Supplementary material for: Salivary microbiome and periodontopathogen/denitrifying bacteria associated with gingivitis and periodontitis in people with type 2-diabetes
Source: F1000Res. 2026 Jan 13;14:297. Originally published 2025 Mar 14. [Version 4] doi: 10.12688/f1000research.161731.4 (PMC12754362; doi:10.12688/f1000research.161731.4)
Supplement: Peer review history

# Reviewer report: Reviewer response for version 4

**DOI:** 10.5256/f1000research.194948.r449913

**Published:** 2026-01-28

**Authors:** Ansam Mahdi Khalel

**Affiliations:** 1 University of Kufa, Najaf, Iraq

I have no further comments to make.

Is the work clearly and accurately presented and does it cite the current literature?

Yes

If applicable, is the statistical analysis and its interpretation appropriate?

Partly

Are all the source data underlying the results available to ensure full reproducibility?

Yes

Is the study design appropriate and is the work technically sound?

Partly

Are the conclusions drawn adequately supported by the results?

Partly

Are sufficient details of methods and analysis provided to allow replication by others?

Yes

Reviewer Expertise:

immunology and microbiology

I confirm that I have read this submission and believe that I have an appropriate level of expertise to confirm that it is of an acceptable scientific standard.

# Reviewer report: Reviewer response for version 4

**DOI:** 10.5256/f1000research.194948.r449917

**Published:** 2026-01-15

**Authors:** Gaetano Isola

**Affiliations:** 1 University of Catania, Catania, Italy

Unfortunately, my previous comments were not still addressed and I am not able to review any advancement compared of the previous version: I suggest that the authors should address my previous comments fully.

Is the work clearly and accurately presented and does it cite the current literature?

Partly

If applicable, is the statistical analysis and its interpretation appropriate?

Yes

Are all the source data underlying the results available to ensure full reproducibility?

Yes

Is the study design appropriate and is the work technically sound?

Yes

Are the conclusions drawn adequately supported by the results?

Partly

Are sufficient details of methods and analysis provided to allow replication by others?

Yes

Reviewer Expertise:

Periodontology, Oral medicine

I confirm that I have read this submission and believe that I have an appropriate level of expertise to state that I do not consider it to be of an acceptable scientific standard, for reasons outlined above.

# Reviewer report: Reviewer response for version 3

**DOI:** 10.5256/f1000research.192091.r439955

**Published:** 2026-01-10

**Authors:** Chandrashekar Janakiram

**Affiliations:** 1 Armita School of Dentistry, Ernakulam, India

Thank you for your detailed rebuttal to the reviewers’ comments. The responses are appropriate and adequately address the concerns raised, resulting in a clear improvement in the scientific rigour of the manuscript.

Is the work clearly and accurately presented and does it cite the current literature?

Yes

If applicable, is the statistical analysis and its interpretation appropriate?

Partly

Are all the source data underlying the results available to ensure full reproducibility?

No source data required

Is the study design appropriate and is the work technically sound?

Yes

Are the conclusions drawn adequately supported by the results?

Yes

Are sufficient details of methods and analysis provided to allow replication by others?

Partly

Reviewer Expertise:

NA

I confirm that I have read this submission and believe that I have an appropriate level of expertise to confirm that it is of an acceptable scientific standard.

# Reviewer report: Reviewer response for version 3

**DOI:** 10.5256/f1000research.192091.r429427

**Published:** 2025-12-30

**Authors:** Susanna Halim

**Affiliations:** 1 Universitas Prima Indonesia, Medan, Indonesia

**ABSTRACT**

 It is known that the main cause of periodontitis is Porphyromonas gingivalis, which serves as the primary pathogen causing inflammation and damage to the supporting tissues of the teeth, and is highly dominant in chronic periodontitis. Meanwhile, Fusobacterium nucleatum is a periopathogen that aids in colonizing plaque and triggering inflammation. In the abstract, the author only demonstrates the relationship between the number of periopathogen bacteria and the number of nitrate-reducing bacteria in saliva microbes, with research results indicating that the greatest abundance of periopathogen bacteria is Porphyromonas. Based on ROC (Receiver Operating Characteristic) analysis, only Fusobacterium spp. and Neisseria  are strong indicators for distinguishing between gingivitis and periodontitis. In the abstract, the author could provide additional descriptions regarding the interaction between Porphyromonas gingivalis and nitrate-reducing bacteria in relation to the damage to the supporting tissues of the teeth (gingivitis or periodontitis), thus serving as an indicator.

**INTRODUCTION**

 In the introduction, the author focuses more on clarifying the periopathogen bacteria and nitrate-reducing bacteria, thereby not concentrating on the interaction between periopathogen bacteria and denitrifying bacteria concerning gingivitis or periodontitis in patients with type 2 diabetes mellitus (T2DM). There is a reciprocal relationship between periodontal disease and type 2 diabetes mellitus. The author needs to add several references in the introduction to strengthen the research objective regarding the relationship between periopathogen bacteria and denitrifying bacteria as factors exacerbating periodontal disease in T2DM patients.

**METHODS**

 In the exclusion criteria, it is necessary to add that samples should not be from one side of the jaw, as chewing on one side can exacerbate the condition of periodontal disease, thus affecting the number of periopathogen and denitrifying bacteria. In the periodontal assessment, it is recommended to perform radiography as a clinical evaluation to support the probing results with the UNC-15 periodontal probe. In the statistical section, the author does not elaborate on the differences among the three groups based on variations between individuals within a single group because the author combines all DNA samples from each group into a single library for sequencing, making statistical tests like ANOVA inappropriate since each group only has one data point on the microbiome profile.

**RESULTS**

 In the research results, there are inaccuracies, particularly in figure 2C, where Fusobacterium spp.  is found in higher proportions compared to other periopathogen bacterial groups, with the highest number in group G2 and the lowest in group G3, whereas it should be lowest in group G1 (without periodontal disease). Porphyromonas gingivalis was found in the highest proportion in group G3 compared to groups G2 and G1. However, Fusobacterium spp. was more numerous than Porphyromonas, whereas Porphyromonas should be more prevalent than Fusobacterium as the main pathogenic bacteria causing inflammation and damage to the supporting tissues of the teeth.

 Figure 2D shows that the proportion of Neisseria, as nitrate-reducing bacteria, is significantly lower in group G1 (without periodontal disease) compared to G2 (gingivitis) and G3 (periodontitis), while the abstract and introduction state that there is a relationship between periopathogen bacteria and nitrate-reducing bacteria; that is, an increase in periopathogen bacteria will lead to a decrease in denitrifying bacteria. Thus, the number of denitrifying bacteria should be lower in group G3 compared to G2 and G1. The author could discuss this matter in the discussion section to explain why this occurs.

**DISCUSSION**

 In the discussion section, the author could add explanations regarding the shift in the proportion of periopathogen bacteria relative to denitrifying bacteria, as well as the effects and reciprocal relationship of non-insulin-dependent T2DM on the severity of periodontal disease and changes in the proportions of periopathogen and denitrifying bacteria.

**CONCLUSION**

 In the conclusion, the author could add the names of the periopathogen and denitrifying bacteria related to the severity of periodontal disease (gingivitis or periodontitis) in T2DM patients, as well as the interaction between periopathogen bacteria and denitrification in gingivitis and periodontitis concerning T2DM.

Is the work clearly and accurately presented and does it cite the current literature?

Yes

If applicable, is the statistical analysis and its interpretation appropriate?

Partly

Are all the source data underlying the results available to ensure full reproducibility?

Yes

Is the study design appropriate and is the work technically sound?

Partly

Are the conclusions drawn adequately supported by the results?

Yes

Are sufficient details of methods and analysis provided to allow replication by others?

Yes

Reviewer Expertise:

biomedical, oral biology, public health, implantology,

I confirm that I have read this submission and believe that I have an appropriate level of expertise to confirm that it is of an acceptable scientific standard.

# Response

**Published:** 2026-01-07

**Authors:** Endang Bachtiar

Response to Reviewer’s (5) comments

 ABSTRACT

 Reviewer’s comments/suggestion (RC/S): In the abstract, the author only demonstrates the relationship between the number of periopathogen bacteria and the number of nitrate-reducing bacteria... based on ROC (Receiver Operating Characteristic) analysis, only Fusobacterium spp. and Neisseria are strong indicators. The author could provide additional descriptions regarding the interaction between P. gingivalis and nitrate-reducing bacteria.

**Author’s Response (AR)**: Thank you for your valuable feedback.

 AR: Thank you for the suggestion. Regarding the ROC analysis, we would like to clarify that following the suggestion from Reviewer 3 in the previous revision stage, we have excluded the ROC analysis from the manuscript. This was decided because our DNA samples were pooled, which limits the statistical validity of individual-based diagnostic performance tests like ROC.

 However, we have addressed your core suggestion by adding a description in the Abstract regarding the interaction between P. gingivalis and nitrate-reducing bacteria. We now highlight that the shift in their relative abundance (the increase of pathogens alongside the decrease of beneficial nitrate-reducers) serves as a key descriptive indicator of periodontal tissue damage in T2DM patients.

 INTRODUCTION

 RC/S: The author focuses more on clarifying the periopathogen and nitrate-reducing bacteria, rather than their interaction concerning gingivitis or periodontitis in T2DM patients. Several references should be added to strengthen the research objective.

**Author’s Response (AR):** We agree. We have revised the Introduction to emphasize the biological interaction between these bacterial groups. Specifically, we have added references explaining how the unique metabolic environment of T2DM patients may disrupt the homeostasis maintained by nitrate-reducing bacteria, thereby facilitating the overgrowth of periodontopathogens. This provides a stronger rationale for our study's focus on their relationship.

 METHODS

 RC/S: In the exclusion criteria, it is necessary to add that samples should not be from one side of the jaw. In the periodontal assessment, radiography is recommended. In the statistical section, the author does not elaborate on individual differences because of the DNA pooling method.

**Author’s Response (AR)**: Thank you for these technical insights.

 1.    Clinical Assessment: We have noted the importance of comprehensive jaw assessment in our protocol description.

 2.    Radiography: We acknowledge that radiographic data were not obtained. We have added this as a study limitation in the Methods section, clarifying that our diagnosis was based on clinical parameters (CAL and Probing Depth) in accordance with the 2017 World Workshop classification.

 3.    Statistics: In response to the DNA pooling method, we have removed all inferential statistics (including the ROC curves previously mentioned). The revised manuscript now adopts a strictly descriptive and exploratory approach, which is more appropriate for pooled sequencing data.

 RESULTS

 RC/S: There are inaccuracies in Figure 2C/D; Fusobacterium spp. was more numerous than Porphyromonas..... the number of denitrifying bacteria (e.g., Neisseria) should be lower in group G3 compared to G2 and G1.

**Author’s Response (AR): **We appreciate the reviewer’s careful review of our data. We have re-verified the taxonomic data from our sequencing results. Figures 2C and 2D have been carefully checked to ensure they accurately represent the relative abundances found in our T2DM cohort. We have also added text to clarify these findings, noting that the observed microbial distribution reflects the specific dysbiotic state of the sampled groups.

 DISCUSSION

 RC/S: The author could add explanations regarding the shift in the proportion of periopathogen bacteria relative to denitrifying bacteria, as well as the effects of T2DM on this relationship.

**Author’s Response (AR):** We have significantly expanded the Discussion section. We now provide a more detailed analysis of the shift from a nitrate-reducing (beneficial) environment to a periodontopathogen-dominant environment. We specifically discuss how T2DM-induced systemic factors (such as oxidative stress) may contribute to this microbial imbalance, exacerbating periodontal tissue destruction.

 CONCLUSION

 RC/S: In the conclusion, the author could add the names of the periopathogen and denitrifying bacteria related to the severity of periodontal disease in T2DM patients.

**Author’s Response (AR): **As suggested, the Conclusion has been revised to be more specific. We now explicitly mention key genera, including Porphyromonas, Fusobacterium, Neisseria, and Rothia, and highlight their shifting interactions as a descriptive hallmark of periodontal disease severity in patients with T2DM.

# Reviewer report: Reviewer response for version 3

**DOI:** 10.5256/f1000research.192091.r439956

**Published:** 2025-12-29

**Authors:** Ansam Mahdi Khalel

**Affiliations:** 1 University of Kufa, Najaf, Iraq

No other comment, I approved the article.

Is the work clearly and accurately presented and does it cite the current literature?

Yes

If applicable, is the statistical analysis and its interpretation appropriate?

Partly

Are all the source data underlying the results available to ensure full reproducibility?

Yes

Is the study design appropriate and is the work technically sound?

Partly

Are the conclusions drawn adequately supported by the results?

Partly

Are sufficient details of methods and analysis provided to allow replication by others?

Yes

Reviewer Expertise:

immunology and microbiology

I confirm that I have read this submission and believe that I have an appropriate level of expertise to confirm that it is of an acceptable scientific standard.

# Reviewer report: Reviewer response for version 3

**DOI:** 10.5256/f1000research.192091.r439958

**Published:** 2025-12-27

**Authors:** Gaetano Isola

**Affiliations:** 1 University of Catania, Catania, Italy

The authors still not fully addressed the previous comments of the first round of revision

Is the work clearly and accurately presented and does it cite the current literature?

Partly

If applicable, is the statistical analysis and its interpretation appropriate?

Yes

Are all the source data underlying the results available to ensure full reproducibility?

Yes

Is the study design appropriate and is the work technically sound?

Yes

Are the conclusions drawn adequately supported by the results?

Partly

Are sufficient details of methods and analysis provided to allow replication by others?

Yes

Reviewer Expertise:

Periodontology, Oral medicine

I confirm that I have read this submission and believe that I have an appropriate level of expertise to state that I do not consider it to be of an acceptable scientific standard, for reasons outlined above.

# Reviewer report: Reviewer response for version 2

**DOI:** 10.5256/f1000research.188726.r426666

**Published:** 2025-11-20

**Authors:** Ansam Mahdi Khalel

**Affiliations:** 1 University of Kufa, Najaf, Iraq

Based on a thorough review of the manuscript, here are comments for each section, focusing on key areas for improvement.

**Abstract**

 The abstract identifies a potential biomarker (the *Fusobacterium-Neisseria* relationship) for distinguishing gingivitis from periodontitis, which is a strong finding. However, it currently states that the G3 (periodontitis) group had the "largest abundance of *Porphyromonas gingivalis*", but fails to contextualize this by comparing it to the other groups or mentioning other key bacterial shifts, which slightly weakens the summary of the overall microbiome changes observed.

**Introduction**

 The introduction effectively establishes the bidirectional link between diabetes and periodontal disease. However, the rationale for focusing specifically on the interplay between periodontopathogens and *denitrifying* bacteria could be strengthened. While denitrifying bacteria are introduced, the text should more explicitly state the central hypothesis: that a disruption in this specific functional group's relationship with pathogens is a key driver of disease progression from gingivitis to periodontitis in the unique metabolic environment of diabetic patients.

**Materials and Methods**

 The methodology of pooling all DNA samples from each of the three groups into a single library for sequencing is a significant limitation that is not adequately addressed. This approach prevents any statistical analysis of inter-individual variation within groups, making it impossible to determine if the observed differences are truly representative of the groups or are skewed by a few outlier individuals. Consequently, the statistical tests mentioned (like Student's t-tests and ANOVA) seem inappropriate as there is only one data point (n=1) for each group's microbiome profile.

**Results**

 The presentation of the heatmap in Figure 4 is critically flawed and misleading. The figure legend claims that "A shift in color toward dark red denotes a higher abundance," but the color key provided clearly indicates the scale represents p-values of statistical significance, not bacterial abundance. This misrepresents the data, as a cell could be dark red (highly significant) but represent a very low abundance. The results section should be revised to present this data accurately, for instance by using a heatmap where color intensity reflects relative abundance and using asterisks to denote statistical significance, rather than conflating the two.

**Discussion**

 The discussion effectively interprets the finding that the *Fusobacterium-Neisseria* interaction may be a strong predictor for the transition from gingivitis to periodontitis. To enhance this section, the authors should propose a more detailed biological mechanism for this specific interaction. For example, they could discuss how *Fusobacterium*'s role as a bridging organism might facilitate the colonization of certain *Neisseria* species that, in the inflammatory diabetic environment, switch from a commensal to a pro-inflammatory role, thereby driving the destructive phase of periodontitis.

**Conclusion**

 The conclusion concisely summarizes that unique relationships between denitrifying and periodontopathic bacteria exist in diabetic patients with periodontal disease. To be more impactful, it should directly state the study's most novel finding as the primary takeaway: that the specific salivary interaction between *Fusobacterium* and *Neisseria* emerges as a promising, non-invasive biomarker for distinguishing between gingivitis and the more severe, irreversible state of periodontitis in individuals with type 2 diabetes.

Is the work clearly and accurately presented and does it cite the current literature?

Yes

If applicable, is the statistical analysis and its interpretation appropriate?

Partly

Are all the source data underlying the results available to ensure full reproducibility?

Yes

Is the study design appropriate and is the work technically sound?

Partly

Are the conclusions drawn adequately supported by the results?

Partly

Are sufficient details of methods and analysis provided to allow replication by others?

Yes

Reviewer Expertise:

immunology and microbiology

I confirm that I have read this submission and believe that I have an appropriate level of expertise to confirm that it is of an acceptable scientific standard.

# Response

**Published:** 2025-11-23

**Authors:** Endang Bachtiar

We sincerely appreciate the reviewer's enthusiasm regarding the biomarker potential of this descriptive observation. **However, due to the methodological constraint of pooling the genomic DNA samples, the calculated Receiver Operating Characteristic (ROC) curve and the Area Under the Curve (AUC) value were statistically invalid.**

**1. Abstract/Conclusion:**

**Action Taken:** We have **removed all ROC analysis** from the manuscript. We have revised the Conclusion to state that this specific microbial feature is a **"promising, descriptive characteristic that warrants further validation"**  for distinguishing disease states, tempering the claim to match the study's revised descriptive scope.

**2. Heatmap:**

**Action Taken:** We have revised the Heatmap ( **Figure 4**) and its caption. The figure now uses **color intensity solely to reflect Relative Abundance** (the descriptive data). **All asterisks and p-values have been completely removed** from the figure, and the caption explicitly states that the data is **strictly descriptive and exploratory**.

**3**. **Introduction:**

**Action Taken:** The final paragraph of the Introduction has been revised to more explicitly state that the central aim is to **descriptively characterize** the disruption of the **oral nitrate-nitrite-NO pathway** in the context of T2DM.

**3**. **Introduction:**

**Action Taken:** We have incorporated a discussion proposing a **hypothetical mechanism** based on descriptive findings, while emphasizing the need for future functional studies to validate this observation.

 4. **Results:**

**Action Taken:** We have completely restructured the Results section to use **strictly descriptive language** for all microbial findings (Rarefaction Curve (Figure 1A), Diversity (Figure 1B, 1C), Phylogenetic Tree ( **Figure 3**), and Heatmap ( **Figure 4**)), while reserving inferential terms only for the clinical and nitrite/nitrate data ( **Figure 5**).

# Reviewer report: Reviewer response for version 2

**DOI:** 10.5256/f1000research.188726.r429433

**Published:** 2025-11-12

**Authors:** Chandrashekar Janakiram

**Affiliations:** 1 Armita School of Dentistry, Ernakulam, India

This cross-sectional study compares pooled salivary microbiomes (ONT MinION whole-16S sequencing) from type-2 diabetes (T2DM) patients grouped by periodontal status (no disease G1, gingivitis G2, periodontitis G3) to explore relationships between classic periodontopathogens and nitrate-reducing/denitrifying bacteria. The authors report higher alpha diversity in diseased groups, greater abundance of *P. gingivalis* in G3, altered nitrate-reducer profiles (notably *Neisseria*, *Rothia*) and an ROC signal (Fusobacterium–Neisseria AUC ≈ 0.93) distinguishing gingivitis from periodontitis. Data and FASTQ files are made available.

**Major concerns**

**Pooled libraries per group — **

1.
  - The methods state that genomic DNA “of each study group’s samples was ligated in equimolar quantities to create a pool of three group libraries for nanopore sequencing” (i.e., one pooled library per group). If true, no individual-level sequence data exist for sequencing analyses, so all downstream diversity statistics (OTU counts, alpha indices, tests comparing groups, correlation heatmaps, ROC analyses) are not valid as inferential tests across subjects. Group-level pooling destroys inter-individual variance and prevents estimation of within-group dispersion, making p-values meaningless. The manuscript must explicitly state whether samples were pooled and justify this choice — but pooling precludes almost every statistical comparison reported. Clarify and, if pooling occurred, rework the paper to present only descriptive, exploratory group-level summaries (no inferential statistics), or (ideally) reprocess/sequences individual samples.
2. **Mismatch between analyses reported and sequencing design / sample unit**
  - Related to (1): the authors report OTU counts (n=1123), alpha diversity tests (Kruskal-Wallis, t-tests), heatmaps of correlations, differential abundance claims and ROC analysis with p-values. These require per-sample counts. The methods must: (a) clearly describe library preparation and whether sequencing reads came from individual barcodes or pooled group barcodes; (b) if barcodes represent individuals, provide per-sample read counts and QC thresholds; (c) if truly pooled, remove inferential testing and reframe conclusions as descriptive.
3. **Bioinformatic preprocessing and compositional data treatment insufficiently described**
  - Important details are missing or ambiguous: the pipeline steps (Dorado basecaller v7.6.8, length filter 200–1500 bp, Kraken2 with ncbi_16s_18s_28s_ITS), thresholds for read inclusion (>5 cumulative reads), normalization / transformation used for relative abundance comparisons, and whether differential abundance tools that account for compositionality (e.g., ANCOM, DESeq2 with appropriate normalization, ALDEx2, Songbird) were used. Microbiome relative-abundance data are compositional and sparse; simple comparisons of raw relative abundances can be misleading. The authors must (a) list exact commands/versions and R packages used; (b) describe normalization, filtering, rarefaction decisions; (c) justify the choice of Kraken2 reference and discuss species-level assignment accuracy with ONT long 16S reads.
4. **Statistical methods: inappropriate tests and missing beta diversity**
  - Authors used Shannon/Simpson for alpha diversity (fine) but then mix Kruskal-Wallis, t-tests and one-way ANOVA in ways that are unclear and possibly inappropriate given data distribution. Beta-diversity analysis (ordination: PCoA/NMDS on Bray-Curtis/UniFrac) is standard to show community shifts between groups and is missing. Differential abundance testing with multiple-test correction is also absent. explicit beta diversity analysis and clearer delineation of which software did which tests.
5. **Study design: selection, grouping and sample size imbalance**
  - The three groups are uneven (G1 N=28, G2 N=54, G3 N=89) and authors acknowledge no formal sample-size calculation. The manuscript must discuss potential bias from imbalance, how confounders (age, sex, HbA1c, medication, smoking) were handled, and whether statistical models adjusted for them.
6. **Clinical periodontal phenotyping lacks radiographic confirmation and full description**
  - Diagnosis relied on clinical CAL/PD and BOP but the manuscript states radiographs were not used. Radiographic bone loss is a standard component of periodontitis staging and its absence weakens case definition; authors must justify omission and discuss how misclassification might affect findings. Also report examiner calibration statistics (kappa or ICC) for PD/CAL measurements 
7. **Biological interpretation overreaches given cross-sectional design**
  - Authors imply mechanistic or predictive roles (e.g., “these features may be crucial markers for early detection”) and use ROC analyses to suggest biomarkers. Given cross-sectional data (and the pooling issue), such claims are premature. Tone down causal/predictive language and limit claims to associations. If ROC analyses are retained, justify them technically (need per-sample data & independent validation).

1. **Figures and legends need rework**
  - Figure legends misplaced earlier (authors note swapping of Fig 3/4 legends). Heatmap (Fig.4) lacks clear legend for colors, significance marks, and clustering annotations. ROC plots must show sample counts, confidence intervals, and cutoffs. Ensure axis labels, units and p-value reporting are consistent.
2. **Nitrite/nitrate measurements — methods and link to sequencing**
  - Griess assay is reported, but authors should specify sample handling (timing of collection relative to probing, storage), limits of detection, and whether nitrite levels correlate at the individual level with microbial taxa (again, requires per-sample data). Address whether saliva was collected before probing (probing can cause bleeding and alter analytes).
3. **Taxonomic claims at species level require caution**
  - Long-read 16S can improve resolution, but Kraken2 on 16S requires careful interpretation; species assignments (e.g., *N. zoodegmatis*, *N. weaveri*) that are normally animal-associated need cautious wording and consideration of misclassification / database contamination. Provide assignment confidence metrics (percent identity, read counts supporting species).
4. **Reproducibility / code and data**
  - Good that FASTQ and subject data are on Figshare; also provide the full analysis scripts (Nextflow / EPI2ME parameters, R scripts) so reviewers can reproduce analyses. 

**Minor **

- Fix typos, duplicated sentences and figure-legend order (authors note they fixed some previously).
- Define abbreviations at first use (NRB, OTU, ONT, Q-score).
- Clarify whether reads are unidirectional (authors say forward or backward) and how that affects assembly/classification.

Is the work clearly and accurately presented and does it cite the current literature?

Yes

If applicable, is the statistical analysis and its interpretation appropriate?

Partly

Are all the source data underlying the results available to ensure full reproducibility?

No source data required

Is the study design appropriate and is the work technically sound?

Yes

Are the conclusions drawn adequately supported by the results?

Yes

Are sufficient details of methods and analysis provided to allow replication by others?

Partly

Reviewer Expertise:

Epidemilogist and ORal Health and Genetics

I confirm that I have read this submission and believe that I have an appropriate level of expertise to confirm that it is of an acceptable scientific standard, however I have significant reservations, as outlined above.

# Response

**Published:** 2025-11-23

**Authors:** Endang Bachtiar

We fully agree with this critical assessment.

**Action Taken:**

 1. **Methods Section:** We have explicitly stated that DNA samples were **pooled in equimolar quantities** and that saliva for nitrite/nitrate measurement was **NOT pooled**.

 2. **Analytical Framework:** We have revised the entire manuscript to adopt a **strictly descriptive and exploratory scope** for all sequencing results.

 3. **ROC Removal:** We have **removed all ROC analysis** from the Abstract, Results, Figures, and Discussion, and the Methods section now explicitly states this omission and the reason.

**Action Taken:** We have **removed all p-values, asterisks, and inferential language** (e.g., 'significantly different') associated with alpha diversity, OTU counts, and taxonomic comparisons throughout the entire manuscript, including all figure captions and legends for Figures 1, 2, 3, and 4.

**Action Taken:** We have included **Table 1: Baseline Demographic and Clinical Characteristics** (on individual-level data). The **Methods** now explicitly state that comparison across groups was performed using **ANOVA for Age** and **Kruskal-Wallis for non-parametric clinical indices** (PD, CAL, % BOP) to ensure statistical validity.

**Action Taken:** This clarification is now explicit in the Methods. We confirm that appropriate inferential statistics (ANOVA/Kruskal-Wallis) were correctly applied to this individual data set and are reported with p-values in the Results ( **Figure 5**).

# Reviewer report: Reviewer response for version 2

**DOI:** 10.5256/f1000research.188726.r420541

**Published:** 2025-10-07

**Authors:** Gaetano Isola

**Affiliations:** 1 University of Catania, Catania, Italy

Unfortunately, the original bias have not been solved. In this version there are some lacks in study design, methodology and results.

Is the work clearly and accurately presented and does it cite the current literature?

Partly

If applicable, is the statistical analysis and its interpretation appropriate?

Yes

Are all the source data underlying the results available to ensure full reproducibility?

Yes

Is the study design appropriate and is the work technically sound?

Yes

Are the conclusions drawn adequately supported by the results?

Partly

Are sufficient details of methods and analysis provided to allow replication by others?

Yes

Reviewer Expertise:

Periodontology, Oral medicine

I confirm that I have read this submission and believe that I have an appropriate level of expertise to state that I do not consider it to be of an acceptable scientific standard, for reasons outlined above.

# Reviewer report: Reviewer response for version 1

**DOI:** 10.5256/f1000research.177804.r406077

**Published:** 2025-09-16

**Authors:** Sonia Nath

**Affiliations:** 1 The University of Adelaide, Adelaide, Australia

Here, the authors report the changes in the salivary microbiome among type 2 diabetes patients, with gingivitis or periodontitis. 

 Introduction:

 Include a brief evidence of the existing literature, are there other studies that have looked at a similar link between diabetes and periodontal disease? Was it conducted on a similar population? What gaps were identified in the previous study? 

 How does this study fills the gaps in the literature. 

 Methods:

 In the inclusion criteria, T2DM patients who were under medication, but still had an unstable condition, were included. 

 How was alveolar bone loss assessed? Did the authors take radiographs? 

 Was sample size calculations considered. There are more number of participants in group 3 than 2 and 1. 

 How many dental examiners were there for the periodontal assessment? If there were more than one periodontist, were they calibrated? Was the score of their consistency. 

 What instrument was used for periodontal assessment? How many site probing was done. 

 Was there any media used to store the saliva samples. Were the samples immediately stored at -80C. 

 Were the saliva samples collected after the probing? Probing can cause bleeding, and this causes inflammation of the gums. Can this have an impact on the saliva samples? How was this mitigated? 

 Any environmental samples collected along with saliva. 

 "After rinsing the mouth"- what was used to rinse the mouth. 

 How was the DNA extraction carried out? Were there any controls used for the DNA extraction process? Similarly, during PCR, were any controls used? 

 Describe the sequencing briefly. What primers were used? Any reference data set used for aligning the sequences. 

 Which packages in R were used for data processing? Mention the names of the packages. 

 Describe clearly for which analysis R Studio was used, and for which analysis GraphPad was used. 

 Results:

 Include a demographic table of the included study participants for each group. 

 Did the authors consider beta diversity analysis? 

 For the realtive abundance table, did the authors apply any filters. Microbiome data are sparse, and did the authors consider transformation of the data or normalisation before analysis for relative abundance? Figure 2B shows only 10 genera. What about the others? Were there any statistical tests performed to compare the differences in the phylum and genera? 

 In Figure 3, there is no legend for the blue colour. What does the * in the blue indicate? Figure 3 is hard to follow. What is A and B. 

 Did the authors consider differential abundance analysis, 

Is the work clearly and accurately presented and does it cite the current literature?

Partly

If applicable, is the statistical analysis and its interpretation appropriate?

No

Are all the source data underlying the results available to ensure full reproducibility?

Yes

Is the study design appropriate and is the work technically sound?

Yes

Are the conclusions drawn adequately supported by the results?

Partly

Are sufficient details of methods and analysis provided to allow replication by others?

Partly

Reviewer Expertise:

Periodontist and microbiolgy researcher

I confirm that I have read this submission and believe that I have an appropriate level of expertise to state that I do not consider it to be of an acceptable scientific standard, for reasons outlined above.

# Response

**Published:** 2025-09-24

**Authors:** Endang Bachtiar

RC#2: Was sample size calculations considered. There are more number of participants in group 3 than 2 and 1. 

 AR: We appreciate the reviewer's insightful assessment of the sample size and participant distribution. We note that no formal statistical procedure was used to establish the number of participants in each category. Instead, we used a consecutive or sequential sampling strategy for a set amount of time (November 2023 to January 2024). Because fewer patients met the requirements for Group 1 (T2DM without periodontal disease) at the time of sampling, this strategy led to an uneven number of participants throughout the groups. We acknowledge that this is a study constraint because it could have an impact on the statistical power of comparisons between groups. However, finding possible microbial markers of illness development was our main goal, and the notable variations in our findings—especially between Group 1 and the other groups—indicate that the sample size was enough for these particular analyses. _We acknowledge that more research with a bigger, more balanced cohort would be helpful to validate our findings, and we have added a sentence to our Limitations section to specifically express this point.

 Reviewer’s#1 comment (RC#1):

 1.The authors should be better specified, at the end of the introduction section, the rationale of the study and the aim of the study.

 AR: Thank you for this important suggestion. Accordingly, at the end of the introduction section, we state the gap of previous studies, as suggested by the Reviewer#2 (yellow highlighted) and the aim of our study (green highlighted).

 2. In the central section, should better clarify inclusions and exclusions criteria of the selected sample.

 AR: Yes, we agree. Accordingly, we have revised the inclusion and exclusion criteria. Many hanks. 

 3. In the Discussion section….better discuss the relationship regarding the by periodontitis in and risk of oxidative stress evolution linked with inflammation in periodontitis patients.

 AR: Thank you for your suggestion. Accordingly, we have added one paragraph to explain the relationships (highlighted in green).

 4. The conclusion should reinforce in light of the discussions.

 AR: We agree. As a result we have revised the conclusion (highlighted in green). Thank you.

 Minor Comments:

 RC#1

 Abstract:

 Better formulate the abstract section by better describing the aim of the study

 Introduction:

 Please refer to major comments

 Discussion

 Please add a specific sentence that clarifies the results obtained in the first part of the discussion

 AR: Thank you for all of the reviewer remarks. Consequently, we have caried out as indicated, the updated version in the respective section. Many thanks.

 Reviewer’s#2  comment (RC#2):

 Introduction section

 Include a brief evidence of the existing literature, are there other studies that have looked at a similar link between diabetes and periodontal disease? Was it conducted on a similar population? What gaps were identified in the previous study? 

 How does this study fills the gaps in the literature. 

 Author’s response (AR)

 Thank you for the suggestion. As a result, we updated the introduction, including the additional research and explaining how it fills in the gaps by concentrating on specific bacterial families (highlighted in yellow). 

 RC#2: Methods:

 In the inclusion criteria, T2DM patients who were under medication, but still had an unstable condition, were included. 

 AR: We appreciate the reviewer's insightful observation. We recognize the possibility of confounding variables being introduced by include patients with different levels of glycemic control, which may be viewed as "unstable." Our main goal, therefore, was to examine a representative sample of people with Type 2 Diabetes, a group in which glycemic control and associated issues frequently differ. Therefore, our findings are more clinically relevant because we included a wide variety of diabetic individuals rather than a highly selected sample. This was minimized by excluding participants who were taking drugs (such as hormones) or had other serious metabolic conditions that would have an adverse effect on the study's results. In the Discussion section, we have included a phrase acknowledging this restriction, which may necessitate additional research in subsequent studies.

 RC#2: How was alveolar bone loss assessed? Did the authors take radiographs? 

 AR: We appreciate the reviewer's wise observation. We recognize that radiographic evaluation is a regular part of a thorough periodontal examination and is an essential tool for assessing alveolar bone loss. However, the main goal of our study was to find non-invasive salivary microbiological markers that could help differentiate between periodontitis and gingivitis. Our patient groups were adequately stratified using clinical characteristics, namely Clinical Attachment Loss (CAL) and probing depth, in accordance with the recognized classifications of periodontal disease. The use of radiographic imaging was deemed outside the purview of this particular study design because our primary focus was on the microbial composition of saliva as a biomarker, a non-invasive sample. _We also think that a more thorough and multifaceted knowledge of the illness progression would be possible with a future study that combines our microbiological findings with radiographic data. To address this problem, we have included a statement in our Limitations section.

 RC#2: Was sample size calculations considered. There are more number of participants in group 3 than 2 and 1. 

 AR: We appreciate the reviewer's insightful assessment of the sample size and participant distribution. We note that no formal statistical procedure was used to establish the number of participants in each category. Instead, we used a consecutive or sequential sampling strategy for a set amount of time (November 2023 to January 2024). Because fewer patients met the requirements for Group 1 (T2DM without periodontal disease) at the time of sampling, this strategy led to an uneven number of participants throughout the groups. We acknowledge that this is a study constraint because it could have an impact on the statistical power of comparisons between groups. However, finding possible microbial markers of illness development was our main goal, and the notable variations in our findings—especially between Group 1 and the other groups—indicate that the sample size was enough for these particular analyses. _We acknowledge that more research with a bigger, more balanced cohort would be helpful to validate our findings, and we have added a sentence to our Limitations section to specifically express this point.

 RC#2:  How many dental examiners were there for the periodontal assessment? If there were more than one periodontist, were they calibrated?

 AR: We thank the reviewer raising this important query. Yes, we accomplished the calibration process and included it in the methods section (highlighted in yellow).

 RC#2:  Was sample size calculations considered. There are more number of participants in group 3 than 2 and 1.

 AR: We appreciate the reviewer's insightful assessment of the sample size and participant distribution. We recognize that no formal statistical procedure was used to establish the number of participants in each category. Rather, we used a sequential sampling method for a particular time frame (November 2023 to January 2024). Because fewer patients met the requirements for Group 1 (T2DM without periodontal disease) at the time of sampling, this strategy led to an uneven number of participants throughout the groups.

 We acknowledge that this is a study constraint because it could have an impact on the statistical power of comparisons between groups. However, finding possible microbial markers of illness development was our main goal, and the notable variations in our findings—especially between Group 1 and the other groups—indicate that the sample size was enough for these particular analyses.

 We acknowledge that more research with a bigger, more balanced cohort would be helpful to validate our findings, and we have added a sentence to our Limitations section to specifically express this point (kindly see the limitation section).

 RC#2: What instrument was used for periodontal assessment? How many sites probing was done

 AR: I value your questions. We employed the UNC-15 probe and the explanation of the probing site, provided in the Methods section.

 RC#2: Was there any media used to store the saliva samples. Were the samples immediately stored at -80C.

 Were the saliva samples collected after the probing? Probing can cause bleeding, and this causes inflammation of the gums. Can this have an impact on the saliva samples? How was this mitigated?

 AR: We did not use any media to store the saliva, and as can be seen in Methods section, “the sample was immediately stores in -80oC until DNA extraction”. Thank you. Saliva samples were collected as well before the probing was carried out. In the Method section, we describe this. 

 RC#2: Any environmental samples collected along with saliva. “After rinsing the mouth"- what was used to rinse the mouth.

 AR: NO, we only collected saliva as oral sample, and 

 we used 0.9% normal saline for about 30 s  as mentioned in the methods section.

 RC#2: How was the DNA extraction carried out? Were there any controls used for the DNA extraction process? Similarly, during PCR, were any controls used? 

 AR: The reviewer's insightful feedback on the PCR and DNA extraction processes is greatly appreciated. We acknowledge that the application of controls is necessary to guarantee the accuracy of the data. As we explained in the Methods, we followed the manufacturer's instructions for the 16S Barcoding Kit and the MonarchTM Genomic DNA purification kit for performing the PCR and DNA extraction processes. The controls were included during the DNA extraction and PCR amplification processes in accordance with the kit's instructions. 

 RC#2: Describe the sequencing briefly. What primers were used? Any reference data set used for aligning the sequences.

 AR: We appreciate your inquiries. Yes, as you can see from the Methods, we have followed the company's instructions for both the alignment and the sequencing process. 

 RC#2: Which packages in R were used for data processing? Mention the names of the packages. 

 Describe clearly for which analysis R Studio was used, and for which analysis GraphPad was used.

 Reviewer’s comment (RC#2):

 For the relative abundance table, did the authors apply any filters. Microbiome data are sparse, and did the authors consider transformation of the data or normalisation before analysis for relative abundance? 

 Author’s response (AR):

 I appreciate your queries. Indeed, we trimmed and filtered them, and we included them in the procedure part under the subheading "DNA extraction, sequencing, and saliva sample collection." 

 RC#2 : Which packages in R were used for data processing? Mention the names of the packages. 

 Describe clearly for which analysis R Studio was used, and for which analysis GraphPad was used. 

 AR: Thank you for these queries, As can be seen in the Methods section, OTUs study was performed using the vegan, ggplot2, and dplyr packages while heatmap was performed using pheatmap and R ColorBrewer packages in R Studio software (version 4.3.2). Moreover,  the Receiver Operating Characteristic (ROC) curves and one-way ANOVA were conducted using GraphPad for the study of salivary nitrite levels.

 Reviewer’s#2 comment and suggestion:

 -Include a demographic table of the included study participants for each group. 

 -Did the authors consider beta diversity analysis? 

 Author’s response.

 I agree. We included a table on characteristics as advised. 

 We recognize that one common method for comparing microbial communities is beta diversity analysis. However, the main goal of our study was to look at the quantity and variety of microorganisms found solely in saliva and no additional oral samples; beta diversity was not taken into account during the analytical process. Thank you very much.

# Reviewer report: Reviewer response for version 1

**DOI:** 10.5256/f1000research.177804.r406078

**Published:** 2025-09-03

**Authors:** Gaetano Isola

**Affiliations:** 1 University of Catania, Catania, Italy

In the manuscript entitled: “Salivary microbiome and periodontopathogen/denitrifying bacteria associated with gingivitis and periodontitis in people with type 2-diabetes" the author aimed to compare the salivary microbiomes of individuals with type 2 diabetes (20–40 years old) who had gingivitis or periodontitis to those who did not. Additionally, were evaluated the relationship between the number of periodontopathogens and the amount of nitrate-reducing bacteria in their salivary microbiome.

 The author found that people with type 2 diabetes mellitus who also have gingivitis or periodontitis exhibit different relationships between periodontopathic and denitrifying bacteria in their salivary microbiome. These features might be essential indicators for early identification and treatment of gingivitis in order to prevent periodontitis.

 Major comments:

 In general, the idea and innovation of this study regards the analysis of the link between mediators, and periodontitis is interesting and novel because the role these aspects in medicine are validated but further studies on this topic could be an innovative issue in this field could be open a creative matter of debate in literature by adding new information. Moreover, there are few reports in the literature that studied this interesting topic with this kind of study design.

 The study was well conducted by the authors; However, there are some concerns to revise that are described below.

 The introduction section resumes the existing knowledge regarding the important factor linked with the relationship between periodontitis and related mediators associated with systemic disease risk.

 However, as the importance of the topic, the reviewer strongly recommends, before a further re-evaluation of the manuscript, to update the literature through read, discuss and cites in the references with great attention all of those recent interesting articles, that helps the authors to better introduce and discuss the relationship and impact of chlorexidine and micro RNA on periodontal tissues and mediators that could impact diabetes amelioration: 1) Ref 1 2) Ref 2

 The authors should be better specified, at the end of the introduction section, the rationale of the study and the aim of the study. In the central section, should better clarify inclusions and exclusions criteria of the selected sample.

 Please better state the results obtained in the abstract.

 The discussion section appears well organized with the relevant paper that support the conclusions, even if the authors should better discuss the relationship regarding the by periodontitis in and risk of oxidative stress evolution linked with inflammation in periodontitis patients. The conclusion should reinforce in light of the discussions.

 In conclusion, I am sure that the authors are fine clinicians who achieve very nice results with their adopted protocol. However, this study, in my view does not in its current form satisfy a very high scientific requirement for indexing in this journal and requests a revision before a futher re-evaluation of the manuscript.

 Minor Comments:

 Abstract:

- Better formulate the abstract section by better describing the aim of the study

Introduction:

- Please refer to major comments

Discussion

- Please add a specific sentence that clarifies the results obtained in the first part of the discussion

Is the work clearly and accurately presented and does it cite the current literature?

Partly

If applicable, is the statistical analysis and its interpretation appropriate?

Yes

Are all the source data underlying the results available to ensure full reproducibility?

Yes

Is the study design appropriate and is the work technically sound?

Yes

Are the conclusions drawn adequately supported by the results?

Partly

Are sufficient details of methods and analysis provided to allow replication by others?

Yes

Reviewer Expertise:

Periodontology, Oral medicine

I confirm that I have read this submission and believe that I have an appropriate level of expertise to confirm that it is of an acceptable scientific standard, however I have significant reservations, as outlined above.

## References

[1] : Impact of periodontal microRNAs associated with alveolar bone remodeling during orthodontic tooth movement: a randomized clinical trial. *Journal of Translational Medicine* .2024;22(1) : 10.1186/s12967-024-05933-x 10.1186/s12967-024-05933-x 39736760 PMC11684316

[2] : Effect of Nonsurgical Mechanical Debridement With or Without Chlorhexidine Formulations in the Treatment of Peri‐Implant Mucositis. A Randomized Placebo‐Controlled Clinical Trial. *Clinical Oral Implants Research* .2025;36(5) : 10.1111/clr.14405 566-577 10.1111/clr.14405 PMC1206690939865359

# Response

**Published:** 2025-09-24

**Authors:** Endang Bachtiar

**Major comment**

**Abstract: **Abstract:

 - Better formulate the abstract section by better    describing the aim of the study.

 - Please better state the results obtained in the abstract.

**AR**: Thank you for these important suggestions. Thus, in the revised version, we have formulated both the study’s aim and the result (highlighted in green).

**RC#1**: ……”introduce and discuss the relationship and impact of chlorhexidine and micro RNA on periodontal tissues and mediators that could impact diabetes amelioration”

**AR**: We thank the reviewer's insightful recommendation. We also agree that including current research on microRNA and chlorhexidine will improve our manuscript by setting our investigation in a more comprehensive scientific framework. Although the microbial associations are the main focus of our work, we have included a few phrases in the Introduction (green highlighted) to recognize their involvement in the intricate interactions between oral and systemic health.

**RC#1**: …..The authors should be better specified, at the end of the introduction section, the rationale of the study and the aim of the study.

**AR: **We agree. this suggestion was also provided the Reviewer#1. Accordingly, we have revised it as suggested. Thank you.
